# Isolation, Structural Assignment of Isoselagintamarlin A from *Selaginella tamariscina* and Its Biomimetic Synthesis

**DOI:** 10.1007/s13659-018-0195-5

**Published:** 2019-01-03

**Authors:** Qin-Feng Zhu, Li-Dong Shao, Xing-De Wu, Jiang-Xin Liu, Qin-Shi Zhao

**Affiliations:** 1 State Key Laboratory of Phytochemistry and Plant Resources in West China, Kunming Institute of Botany, Chinese Academy of Sciences, Kunming, 650201 People’s Republic of China; 2 University of Chinese Academy of Sciences, Beijing, 100049 People’s Republic of China

**Keywords:** *Selaginella tamariscina*, Selaginellin, Biomimetic synthesis, Isoselagintamarlin A

## Abstract

**Abstract:**

Isoselagintamarlin A (**1**), a selaginellin analogue featured a rare benzofuran unit, was isolated from *Selaginella tamariscina*. Its complete structural assignment was established through a combination of high-field NMR technique and biomimetic synthesis. Notably, isoselagintamarlin A (**1**) was successfully synthesized via sequential oxidations and intramolecular cyclization.

**Graphical Abstract:**

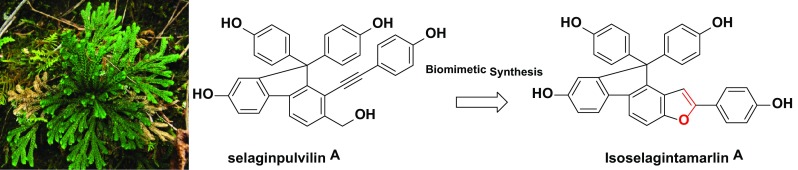

**Electronic supplementary material:**

The online version of this article (10.1007/s13659-018-0195-5) contains supplementary material, which is available to authorized users.

## Introduction

**Fig. 1:**
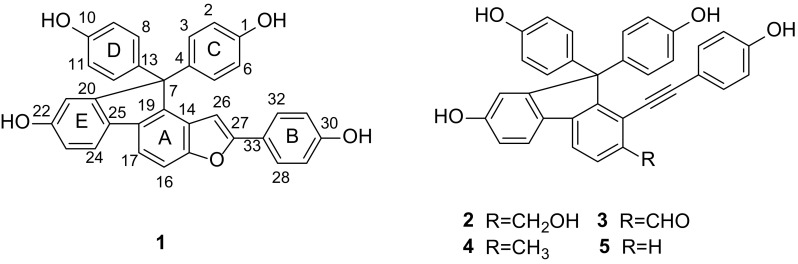
Structures of compounds **1**-**5**

The selaginellin derivatives, isolated from the genus *Selaginella*, are a family of natural pigments characterized by acetylenic link and *p*-quinone methide functionalities [1–4]. The isolation and synthesis of selaginellin and its analogues have attracted tremendous attentions recently due to their fascinating structures and a wide range of biological activities [5–11]. *Selaginella tamariscina*, a qualified species listed in the Chinese Pharmacopoeia, has been used in Traditional Chinese Medicine for the treatment of amenorrhea, dysmenorrhea, and traumatic injury, and has been reported containing some selaginellin derivatives [12]. In an earlier study towards the discovery of structurally interesting and bioactive natural products, several selaginellin analogues with good inhibitory activities against BACE1 were previously reported from *S. tamariscina* [13]. In the current study, a further phytochemical investigation on this plant led to the isolation of an unprecedented benzofuran-type selaginellin derivative named isoselagintamarlin A (**1**), along with four known analogues, selaginpulvilins A-D (**2**-**5**) (Fig. 1). Herein, we present the isolation and complete structural assignment of isoselagintamarlin A (**1**) based on the combination of high-field NMR techniques and the first biomimetic synthesis.

## Results and Discussion

The air-dried and powdered whole plants of *S. tamariscina* were extracted with 70% EtOH for three times. Further column chromatography (CC) over MCI gel, normal-phase silica gel, Sephadex LH-20 and semi-preparative HPLC led to the isolation of one new selaginellin derivative (**1**), and four known ones (**2-5**).

Compound (**1**) was obtained as yellow oil. Its molecular formula was determined as C_33_H_22_O_5_ by HR-EI-MS with an ion peak at *m/z* 498.1454 [M]^+^ (calcd 498.1467), which indicated 23 degrees of unsaturation. The IR spectrum exhibited absorption bands for hydroxy (3427 cm^−1^) and aromatic (1612 and 1507 cm^−1^) functionalities. Some aromatic proton signals at 6.80–7.00 ppm overlapped each other in the ^1^H NMR spectrum obtained from a 600 MHz spectrometer (Fig. S1, Electronic supplementary material). In order to characterize those key signals, the NMR experiments of compound **1** were carried out in an 800 MHz spectrometer, and we were pleased to find that the overlapping proton signals were distinguishable (Fig. S2, Electronic supplementary material). The ^1^H NMR spectrum of **1** (Table 1) showed the signals of three *p*-substituted phenyl groups (two were overlapped) [*δ*_H_ 6.70 (4H, d, *J* = 8.9 Hz, H-2, 6, 9, 11), 7.08 (4H, d, *J* = 8.9 Hz, H-3, 5, 8, 12), 6.90 (2H, d, *J* = 8.8 Hz, H-29, 31), 7.72 (2H, d, *J* = 8.8 Hz, H-28, 32)], a 1,2,4-trisubstituted benzene ring [*δ*_H_ 6.83 (1H, d, *J* = 8.1, 2.3 Hz, H-23), 6.91 (1H, s, H-21), 7.65 (1H, d, *J* = 8.1 Hz, H-24)], a 1,2,3,4-tetrasubstituted phenyl ring [*δ*_H_ 7.49 (1H, d, *J* = 8.3 Hz, H-16), 7.66 (1H, d, *J* = 8.3 Hz, H-17)], and an olefinic proton [*δ*_H_ 6.88 (1H, br s, H-26)]. The ^13^C NMR data (Table 1) in combination with DEPT spectra exhibited 33 carbon signals that were ascribable to an alkenyl (*δ*_C_ 157.5, C-27; 99.0, C-26), three *p*-phenyl groups (two were overlapped), two polysubstituted phenyl rings, and an *sp*^3^ quaternary carbon (*δ*_C_ 65.1, C-7). The aforementioned information was indicative of the skeleton of a selaginpulvilin derivative [7], with the structural variations occuring on the alkynyl and formyl parts.

**Table 1:** NMR data of compound **1** (*δ* in ppm, *J* in Hz)

Position	*δ*_H_^a^	*δ*_C_^b^	HMBC H → C^b^
1/10		157.0	
2/6/9/11	6.70 (d, *J* = 8.9)	115.7	1, 3, 4
3/5/8/12	7.08 (d, *J* = 8.9)	130.4	1, 2, 7
4/13		136.2	
7		65.1	
14		127.3	
15		155.3	
16	7.49 (d, *J* = 8.3)	110.8	14, 15, 18
17	7.66 (d, *J* = 8.3)	115.8	19, 25
18		135.8	
19		144.0	
20		156.0	
21	6.91 (s)	113.5	7, 22, 23, 25
22		157.8	
23	6.83 (dd, *J* = 8.1, 2.3)	115.3	21, 22, 25
24	7.65 (d, *J* = 8.1)	121.0	18, 20, 22
25		133.1	
26	6.88 (br s)	99.0	14, 15, 27
27		157.5	
28/32	7.72 (d, *J* = 8.8)	127.4	27, 29, 30
29/31	6.90 (d, *J* = 8.8)	116.6	30, 33
30		159.2	
33		122.7	

Assignments confirmed by DEPT, HSQC, HMBC, COSY, and ROESY NMR experiments

^a^Compound was recorded in acetone-*d*_6_ at 800 MHz

^b^Compound was recorded in acetone-*d*_6_ at 200 MHz

**Fig. 2:**
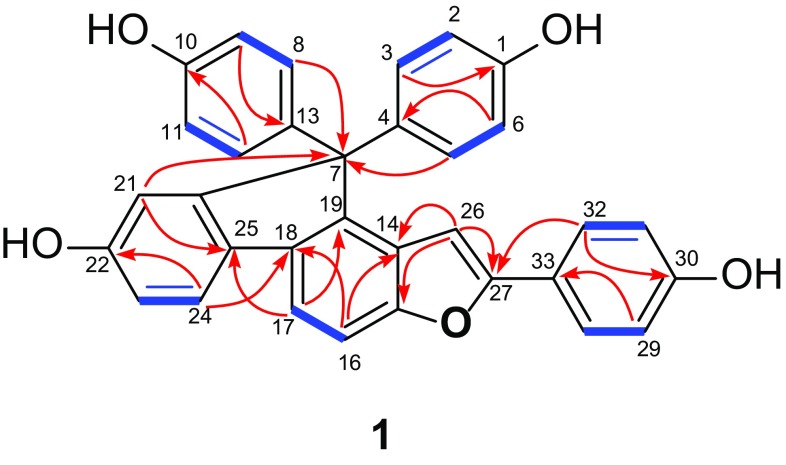
Selected ^1^H-^1^H COSY (  ) and HMBC (  ) correlations of **1**

The connectivities of these benzene rings, the quaternary carbon and the alkenyl could be well interpreted by 2D NMR analysis (Fig. 2). Two symmetrical *para*-substituted benzene rings were located at C-7, as demonstrated by HMBC correlations of H-3, H-5, H-8, and H-12 with C-7. The HMBC correlations from H-17 to C-25 (*δ*_C_ 133.1) and from H-24 to C-18 (*δ*_C_ 135.8) suggested two multisubstituted benzene rings was linked via C-18/C-25 bond. In addition, ring E was further linked to C-7 on the basis of the HMBC correlation from H-21 to C-7 of the fluorene. Further observation of the cross-peaks between the olefinic proton H-26 and C-14 (*δ*_C_ 127.3), C-15 (*δ*_C_ 155.3), and C-27 (*δ*_C_ 157.1) placed a styryl group at C-14. Moreover, the 4-hydroxylphenyl group was linked to C-27 by HMBC correlations of H-28 and H-32 with C-27. Due to the five phenyl rings, a fluorene core, a double bond only expended 22 of the 23 degrees of unsaturation, the remaining unsaturation unit required that **1** had one more ring than that of selaginpulvilin, and the severely downfield-shifted *sp*^2^ carbon at C-27 led to the construction of a furan ring between C-15 and C-27. On the basis of the above evidence, the gross structure of **1** with a 2-(4-hydroxyphenyl)-benzofuran unit was proposed (Fig. 1), which was fully consistent with its molecular composition, and represented a new skeleton for the selaginpulvilins.

**Scheme 1:**
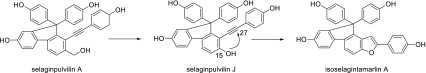
Plausible biogenetic formation of **1**

Isoselagintamarlin A represents a hitherto unknown selaginellin skeleton, based on the cooccurrence of compounds **2**-**5**, a plausible biogenetic pathway for **1** was proposed (Scheme 1). Selaginpulvilin A (**2**), the major component, was considered as the precusor. In brief, **2** underwent sequential oxidation to form selaginpulvilin J. The key step in this proposal was that the hydroxy group at C-15 attacked the triple bond to form a stable furan ring of isoselagintamarlin A (**1**).

Since no direct HMBC correlations were available to the new ring, as well as the limited amount of **1**, its single crystals could not be obtained. Taken together, the structure of **1** remains ambiguous. We thus decided to carry out a biomimetic semisynthesis of **1**, which can not only unequivocally confirm the complete structure but also provide sufficient quantities for further bioactiviy studies.

**Scheme 2:**
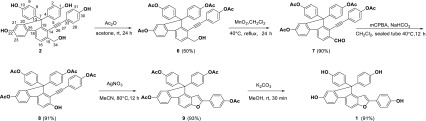
Biomimetic semisynthesis structures of compound **1**

As outlined in Scheme 2, selaginpulvilin A (**2**), the major component, was considered as the precusor of **1**. Selaginpulvilin A (**2**) was first converted to its acetylated (Ac_2_O without base in acetone) derivative (**6**), which then reacted with MnO_2_ at 40 °C for 24 h to generate aldehyde 7. Next, compound **7** was directly converted to phenol **8** under the condition of *m*CPBA/NaHCO_3_ through the Baeyer–Villiger oxidation [14] reaction in 91% yield. According to the hypothetical biogenetic pathway of **1** (Scheme 1), compound **9** could be generated from a 5-*exo*-*dig* cyclization of **8** in the presence of catalytic AgNO_3_ in 93% yield. Finally, **9** was treated with K_2_CO_3_ to provide the target molecule **1**. The spectroscopic data (^1^H, ^13^C NMR and HR-ESI–MS analysis) of the synthetic compound were identical to those of natural isoselagintamarlin A (**1**), which further secured the structure of **1**. Furthermore, it was reported that the conversion of selaginpulvilin A to selaginpulvilins B, F and H were unsuccessful by the reason that there was no oxidation of hydroxy group presented in trimethyl-selaginpulvilin A [9]. In the biomimetic semisynthesis tetraacetylated-selaginpulvilin A could be transformed into tetraacetylated-selaginpulvilin B, which provided an opportunity for the synthesis of other members of this family of natural products.

The known compounds were identified as selaginpulvilins A-D (**2**-**5**) by comparison of their spectroscopic and physical data with those in the literature [7].

## Experimental

### General

IR spectra were obtained on a Tenor 27 spectrometer with KBr pellets. ^1^H and ^13^C NMR spectra were performed on AVANCE III-600 and AV 800 spectrometers with TMS as an internal standard (Bruker, Karlsruhe, Germany). ESIMS were run on an Agilent 6540 Q-TOF spectrometer (Agilent, Palo Alto, CA, USA). HR-EI-MS were run on an Shimadzu UPLC-IT-TOF spectrometer. HR-ESI-MS were measured using Agilent G6230 TOF MS (Agilent, Palo Alto, CA, USA). Semi-preparative HPLC was performed on an Agilent 1260 apparatus equipped with a diode-array detector and a Zorbax SB-C18 (Agilent, 9.4 mm × 25 cm) column. Column chromatography (CC) was performed using MCI gel (CHP 20P, 75–150 mm; Mitsubishi Chemical Corporation, Tokyo, Japan), silica gel (100–200 or 200–300 mesh, Qingdao Marine Chemical Co. Ltd., Qingdao, China) and Sephadex LH-20 (Amersham Pharmacia Biotech, Sweden). Thin-layer chromatography (TLC) was carried out on silica gel GF_254_ on glass plates (Qingdao Marine Chemical Inc.) and spots were visualized by heating silica gel plates sprayed with 10% H_2_SO_4_ in EtOH. All reactions sensitive to air or moisture were carried out under argon or nitrogen atmosphere in dry and freshly distilled solvents under anhydrous conditions, unless otherwise noted.

### Plant Material

The entire plant of *S. tamariscina* used in this study was purchased from kunming Chinese herbal medicine professional market, Kunming, Yunnan Province, People’s Republic of China in 2014 and identified by Prof. Xiao Cheng of the Kunming Institute of Botany, Chinese Academy of Sciences. A voucher specimen (No. 20140608P01) has been deposited at the state key Laboratory of Phytochemistry and Plant Resources in West China, Kunming Institute of Botany, Chinese Academy of Sciences, People’s Republic of China.

### Extraction and Isolation

The air-dried powder of the entire plants of *Selaginella tamariscina* (11 kg) was extracted three times with 70% EtOH (3 × 35 L) at room temperature for 72 h, which was then concentrated in vacuo to give deposition portion (450 g). The deposition were chromatographed over a reversed-phase preparative MPLC (MCI) column eluting with a gradient mobile phase (MeOH/H_2_O, 5% → 95%, v/v) to give five fractions A-E. Fraction C (60 g) was chromatographed over a silica gel column (CHCl_3_/MeOH, 100:1-0:1) to get five fractions (I–V), based on their TLC characteristics. Fraction III (19 g) was further separated by silica gel column eluted with CHCl_3_/MeOH (20:1 → 0:1, v/v) to give five fractions (III-A to III-E). Fraction III-D (2.5 g) was further separated by Sephadex LH-20 column (MeOH) and then purified by semi-preparative HPLC using 35% MeCN/H_2_O (flow rate = 5 mL min^−1^) to afford **2** (380 mg, *t*_R_ = 24.7 min) and **5** (65 mg, *t*_R_ = 28.3 min), respectively. Fraction III-C (1.8 g) was subjected to silica gel column chromatography (petroleum ether/acetone, 6:4, v/v) to give two sub-fractions (III-Ca to III-Cb). Fraction III-Ca (210 mg) was separated by semi-preparative HPLC (32% MeCN/H_2_O, flow rate = 5 mL min^−1^) to yield compound **1** (1.6 mg, *t*_R_ = 27.5 min). Fraction III-Cb (480 mg) was isolated repeatedly by Sephadex LH-20 gel column (MeOH) and then separated by semi-preparative HPLC (42% MeCN/H_2_O, flow rate = 5 mL min^−1^) to obtain **3** (8 mg, *t*_R_ = 14.1 min) and **4** (18 mg, *t*_R_ = 17.5 min).

#### Isoselagintamarlin A (**1**)

Yellow oil; IR (KBr) υ_max_ 3427, 1612, 1507, 1427 and 794 cm^−1^. UV (MeOH) λ_max_ (log ε) 314 (4.34), 284 (4.25), 204 (4.25). ^1^H-NMR (800 MHz, acetone-*d*_6_) and ^13^C-NMR (200 MHz, acetone-*d*_6_), see Table 1. HR-EI-MS *m/z* 498.1454 [M]^+^ (calcd for C_33_H_22_O_5_, 498.1467).

### Semisynthesis and Characterization

Tetraacetylated-selaginpulvilin A (**6**). A sample of acetic anhydride (274.5 *u*L) was added to a solution of **2** (248 mg) in dry acetone (15 mL), and the mixture was stirred at rt until the starting material was consumed (TLC analysis). After solvent removing, the residue was purified by flash column chromatography on silica gel (petroleum ether/acetone = 2:1, v/v) to give acetylation product **6** as a yellow oil (163 mg, 50% yield).^1^H-NMR (CDCl_3_, 600 MHz) *δ*_H_ 7.75 (1H, d, *J *= 8.0 Hz), 7.74 (1H, d, *J* = 8.0 Hz), 7.55 (1H, d, *J* = 8.0 Hz), 7.29 (4H, d, *J* = 8.7 Hz), 7.14 (1H, d, *J* = 8.0 Hz), 7.04 (2H, d, *J* = 8.5 Hz), 7.02 (1H, s), 7.01 (2H, d, *J* = 8.5 Hz), 6.90 (4H, d, *J* = 8.7 Hz), 4.87 (2H, s), 2.30 (3H, s), 2.25 (9H, s); ^13^C-NMR (CDCl_3_, 150 MHz) *δ*_C_ 169.3 (s), 169.1 (s), 154.0 (s), 152.0 (s), 150.9 (s), 150.8 (s), 149.5 (s), 142.5 (s), 139.5 (s), 139.1 (s), 136.5 (s), 132.5 (d), 130.0 (d), 127.4 (d), 122.0 (d), 121.4 (d), 121.0 (d), 120.5 (d), 120.3 (d), 120.1 (s), 119.1 (s), 119.0 (d), 101.0 (s), 85.5 (s), 65.4 (s), 63.9 (t), 21.1 (q); HR-ESI-MS *m/z*: 703.1941 [M + Na]^+^ (calc. for C_42_H_32_O_9_Na, 703.1939).

Tetraacetylated-selaginpulvilin B (**7**). Compound **6** (150 mg), activated MnO_2_ (191.9 mg) and DCM (25 mL) were placed in a 75 mL thick walled glass pressure tube. The tube was sealed and the solution was stirred at 40 °C for 24 h. After cooling to room temperature, the mixture was filtered, evaporated under vacuum, and the residue was purified by flash column chromatography on silica gel (petroleum ether/acetone = 4:1, v/v) to give aldehyde **7** as a yellow oil (135 mg, 90% yield). ^1^H-NMR (CDCl_3_, 600 MHz) *δ*_H_ 10.5 (1H, *s*), 8.03 (1H, d, *J* = 8.0 Hz), 7.82 (1H, d, *J* = 8.0 Hz), 7.81 (1H, d, *J* = 8.0 Hz), 7.28 (4H, d, *J* = 8.8 Hz), 7.18 (1H, dd, *J* = 8.0, 2.0 Hz), 7.06 (1H, br s), 7.05 (2H, d, *J* = 8.7 Hz), 7.02 (2H, d, *J* = 8.7 Hz), 6.94 (4H, d, *J* = 8.7 Hz), 2.28 (3H, s), 2.24 (9H, s); ^13^C-NMR (CDCl_3_, 150 MHz) *δ*_C_ 191.2 (d), 169.3 (s), 169.0 (s), 155.4 (s), 152.4 (s), 152.0 (s), 151.2 (s), 149.8 (s), 145.5 (s), 138.2 (s), 135.4 (s), 135.3 (s), 132.6 (d), 129.9 (d), 128.1 (d), 124.3 (d), 122.1 (d), 121.9 (d), 121.7 (d), 121.1 (d), 120.2 (s), 119.4 (s), 119.2 (d), 103.4 (s), 83.7 (s), 65.4 (s), 21.1 (q); HR-ESI–MS *m/z*: 701.1780 [M + Na]^+^ (calc. for C_42_H_30_O_9_Na, 701.1782).

Tetraacetylated-selaginpulvilin J (**8**). To a stirred solution of **7** (100 mg) in dry DCM (15 mL), NaHCO_3_ (24.8 mg) and *m*CPBA (38.2 mg) was added. The mixture was sealed and stirred at 40 °C for 12 h, then washed with saturated NaHCO_3_ solution for three times. After removal of solvents, the residue was purified by flash column chromatography on silica gel (petroleum ether/acetone = 4:1, v/v) to give phenol **8** as a yellow oil (89 mg, 91% yield). ^1^H-NMR (CDCl_3_, 600 MHz) *δ*_H_ 7.64 (1H, d, *J* = 8.3 Hz), 7.61 (1H, d, *J* = 8.3 Hz), 7.26 (4H, d, *J* = 8.8 Hz), 7.09 (2H, d, *J* = 8.6 Hz), 7.08–7.10 (1H, m), 7.04 (2H, d, *J* = 8.6 Hz), 7.02 (1H, d, *J* = 8.3 Hz), 6.98 (1H, d, *J* = 2.0 Hz), 6.90 (4H, d, *J* = 8.8 Hz), 2.28 (3H, s), 2.24 (6H, s), 2.22 (3H, s); ^13^C-NMR (CDCl_3_, 150 MHz) *δ*_C_ 169.4 (s), 169.3 (s), 169.1 (s), 156.8 (s), 153.1 (s), 152.3 (s), 151.0 (s), 150.0 (s), 149.6 (s), 139.3 (s), 137.0 (s), 132.6 (d), 132.5 (s), 130.0 (d), 122.0 (d), 121.9 (d), 121.3 (d), 121.0 (d), 119.6 (d), 119.5 (s), 118.9 (d), 114.5 (d), 107.8 (s), 102.4 (s), 81.7 (s), 65.2 (s), 21.1 (q); HR-ESI–MS *m/z*: 705.1524 [M + K]^+^ (calc. for C_41_H_30_O_9_K, 705.1521).

Tetraacetylated-isoselagintamarlin A (**9**). To a stirred solution of **8** (76 mg) in MeCN (15 mL), AgNO_3_ (9.7 mg) was added. The mixture was stirred at 80 °C for 12 h and the solvent was evaporated under vacuum. The residue was diluted with H_2_O and extracted with EtOAc (10 mL × 3), dried over Na_2_SO_4_. The solvent was evaporated under vacuum and the residue was purified by flash column chromatography on silica gel (petroleum ether/acetone = 5:1, v/v) to afford compound **9** as a yellow oil (71 mg, 93% yield). ^1^H-NMR (aceton-*d*_6_, 600 MHz) *δ*_H_ 7.93 (2H, d, *J* = 8.6 Hz), 7.91–7.93 (1H, m), 7.90 (1H, d, *J* = 8.4 Hz), 7.67 (1H, d, *J* = 8.4 Hz), 7.32 (4H, d, *J* = 8.8 Hz), 7.28 (1H, d, *J* = 2.0 Hz), 7.23 (1H, s), 7.21 (2H, d, *J* = 8.6 Hz), 7.18 (1H, dd, *J* = 8.2, 2.0 Hz), 7.04 (4H, d, *J* = 8.8 Hz), 2.26 (3H, s), 2.21 (3H, s), 2.20 (6H, s); ^13^C-NMR (aceton-*d*_6_, 150 MHz) *δ*_C_ 169.7 (s), 169.5 (s), 169.5 (s), 157.0 (s), 156.3 (s), 154.1 (s), 152.4 (s), 151.4 (s), 151.0 (s), 144.0 (s), 141.8 (s), 138.8 (s), 135.1 (s), 130.2 (d), 128.3 (s), 127.1 (s), 127.0 (d), 123.2 (d), 122.5 (d), 122.5 (d), 121.1 (d), 120.1 (d), 117.7 (d), 112.0 (d), 101.1 (d), 67.3 (s), 20.9 (q); HR-ESI–MS *m/z*: 705.1531 [M + Na]^+^ (calc. for C_41_H_30_O_9_Na, 705.1521).

Isoselagintamarlin A (**1**) prepared from biomimetic semisynthesis. The mixture of **9** (56 mg) and K_2_CO_3_ (69.7 mg) in MeOH (15 mL) was stirred at room temperature for 30 min, and the solvent was evaporated under vacuum. The residue was diluted with H_2_O and extracted with EtOAc (10 mL × 3), dried over Na_2_SO_4_. The solvent was evaporated under vacuum and the residue was purified by flash column chromatography on silica gel (petroleum ether/acetone = 2:1, v/v) to afford **1** as a yellow oil (38 mg, 91% yield). The NMR data of this synthetic compound are consistent with those of this compound isolated from plants, see Table 1; HR-ESI-MS *m/z*: 497.1380 [M − H]^−^ (calc. 497.1394).

## Electronic supplementary material

Below is the link to the electronic supplementary material.

Supplementary material 1 (DOCX 3034 kb)
