# Supplementary material for: Isolation, Structural Assignment of Isoselagintamarlin A from Selaginella tamariscina and Its Biomimetic Synthesis
Source: Nat Prod Bioprospect. 2019 Jan 3;9(1):69–74. doi: 10.1007/s13659-018-0195-5 (PMC6328423; doi:10.1007/s13659-018-0195-5)

**Isolation, Structural Assignment of Isoselagintamarlin A from *Selaginella tamariscina* and Its Biomimetic Synthesis**

Qin-Feng Zhu^1,2^·Li-Dong Shao^1^·Xing-De Wu^1^·Jiang-Xin Liu^1^·Qin-Shi Zhao^1^

- Qin-Shi Zhao

qinshizhao@mail.kib.ac.cn

^1^ State Key Laboratory of Phytochemistry and Plant Resources in West China, Kunming Institute of Botany, Chinese Academy of Sciences, Kunming 650201, People’s Republic of China.

^2^ University of Chinese Academy of Sciences, Beijing 100049, People’s Republic of China

**Table of Contents**

| **Figure** | **Contents** |
| --- | --- |
| 1-9 | NMR, UV, IR, HR-EI-MS spectra of isoselagintamarlin A (**1**) |
| 10-15 | NMR and HR-ESI-MS of *tetra*-acetylated selaginpulvilin A (**6**) |
| 16-21 | NMR and HR-ESI-MS of *tetra*-acetylated selaginpulvilin B (**7**) |
| 22-27 | NMR and HR-ESI-MS of *tetra*-acetylated selaginpulvilin J (**8**) |
| 28-33 | NMR and HR-ESI-MS of *tetra*-acetylated isoselagintamarlin A (**9**) |
| 34-36 | NMR and HR-ESI-MS of isoselagintamarlin A (**1**) (synthetic product) |

**Figure S1.** ^1^H NMR spectrum of isoselagintamarlin A (**1**) in acetone-*d*_6_ (600 MHz)

**
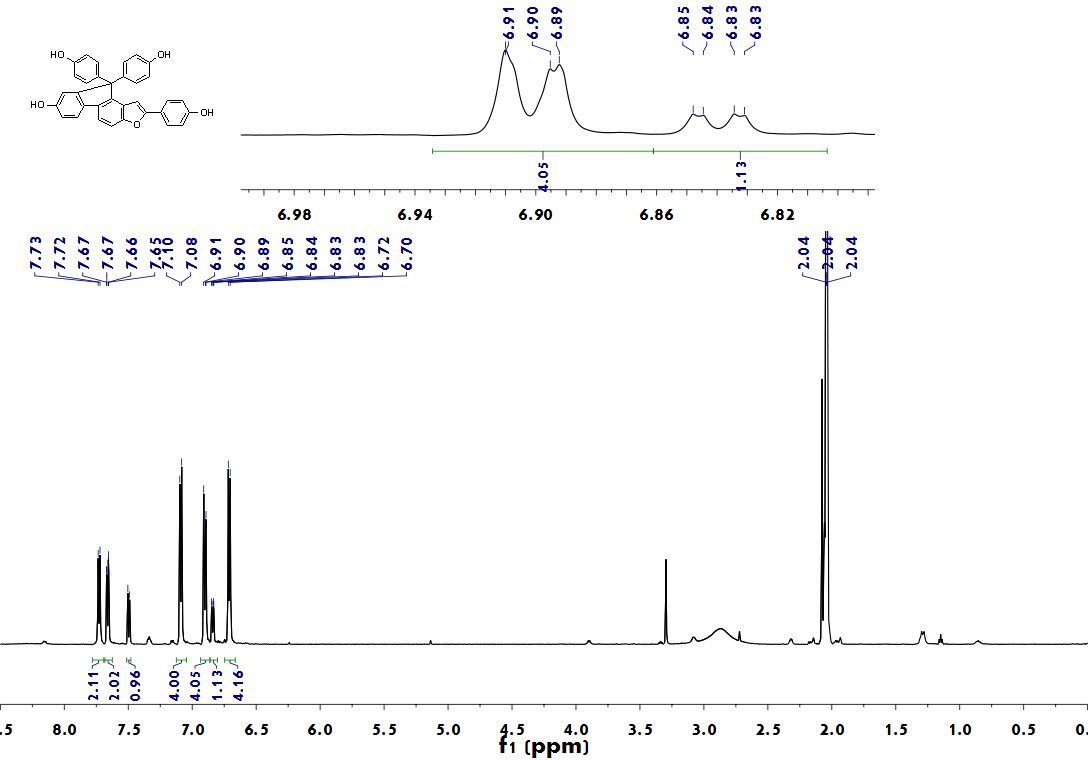
**

**Figure S2.** ^1^H NMR spectrum of isoselagintamarlin A (**1**) in acetone-*d*_6_ (800 MHz)

Natural product


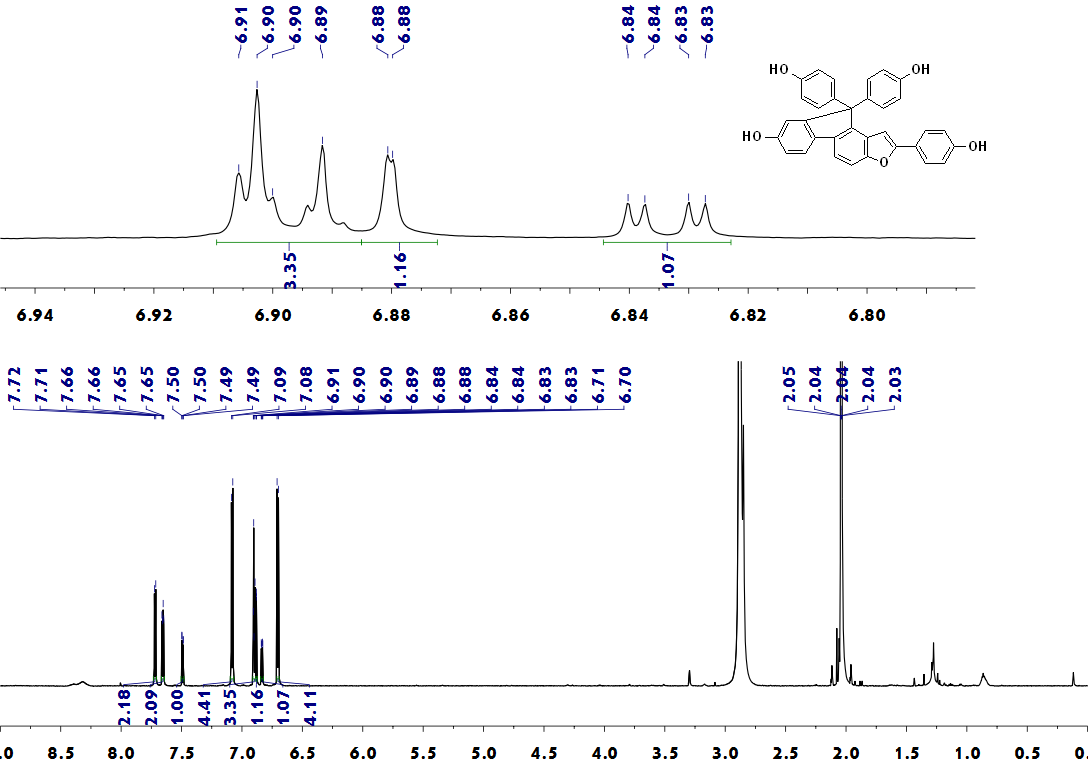


**Figure S3.** ^13^C NMR spectrum of isoselagintamarlin A (**1**) in acetone-*d*_6_ (200 MHz)

Natural product


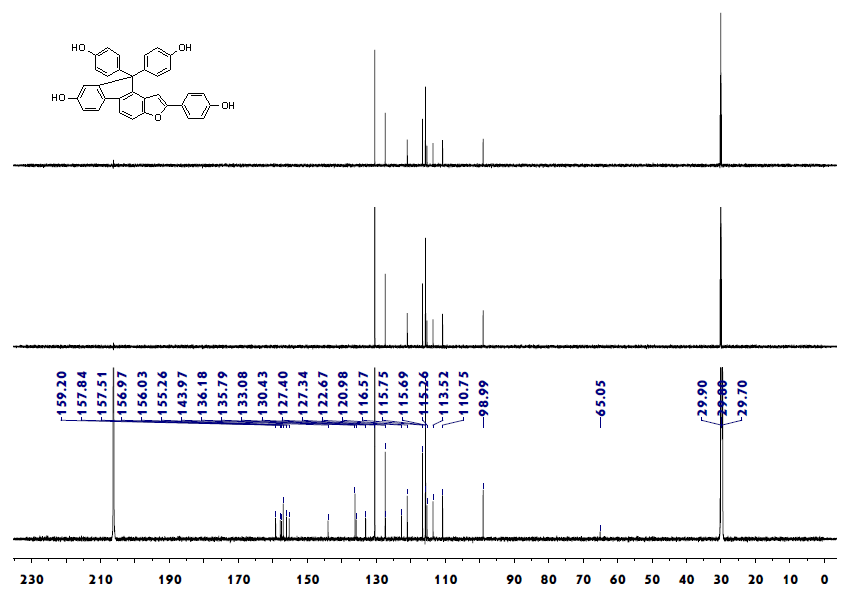


Synthetic product


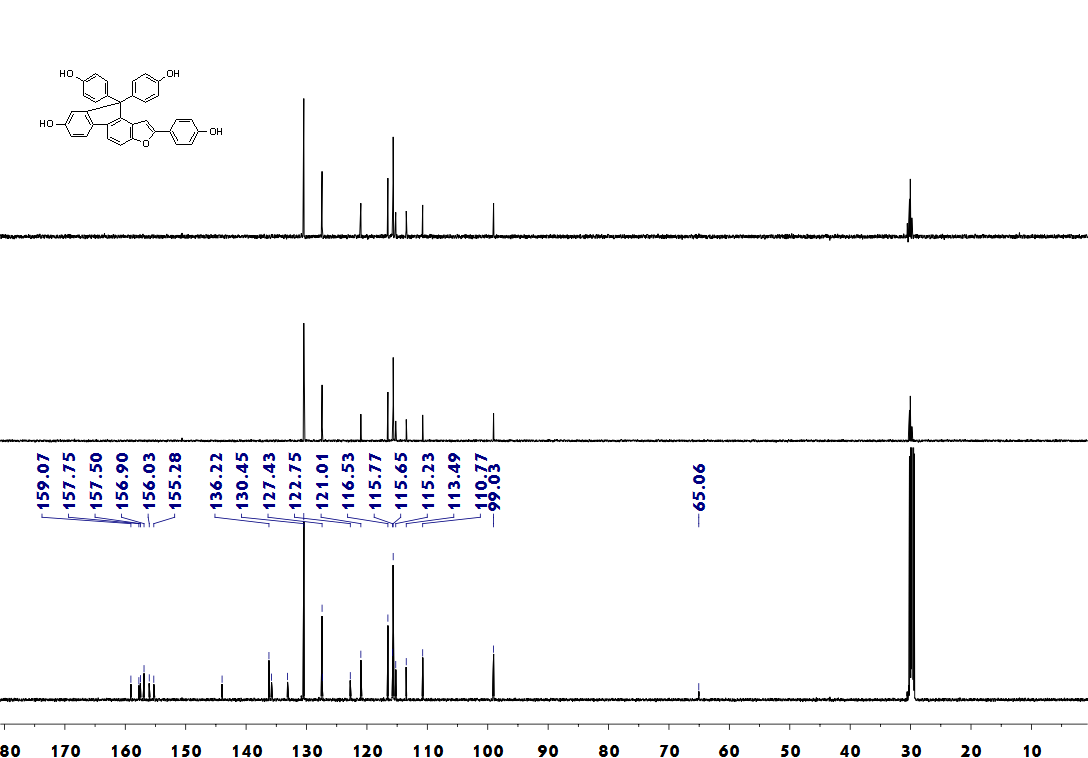


**Figure S4.** ^1^H-^1^H COSY spectrum of isoselagintamarlin A (**1**)


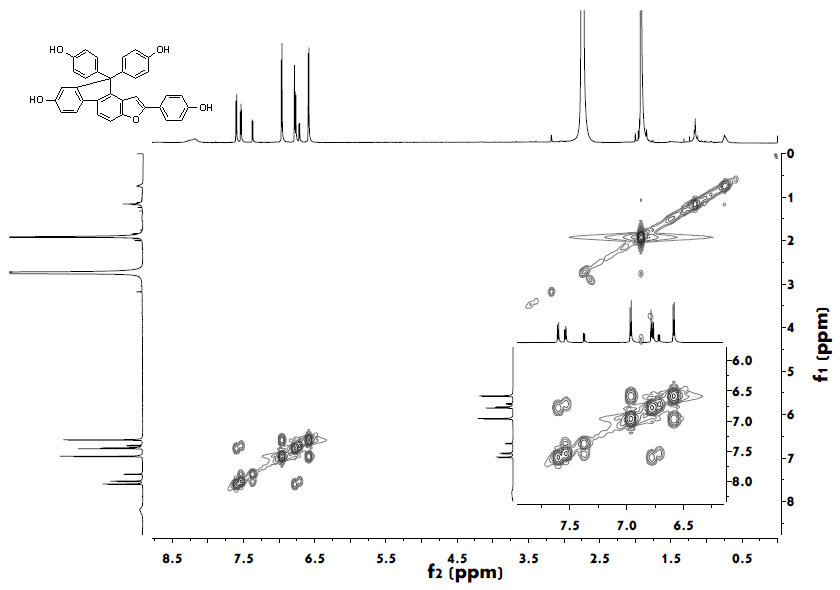


**Figure S5.** HSQC spectrum spectrum of isoselagintamarlin A (**1**)


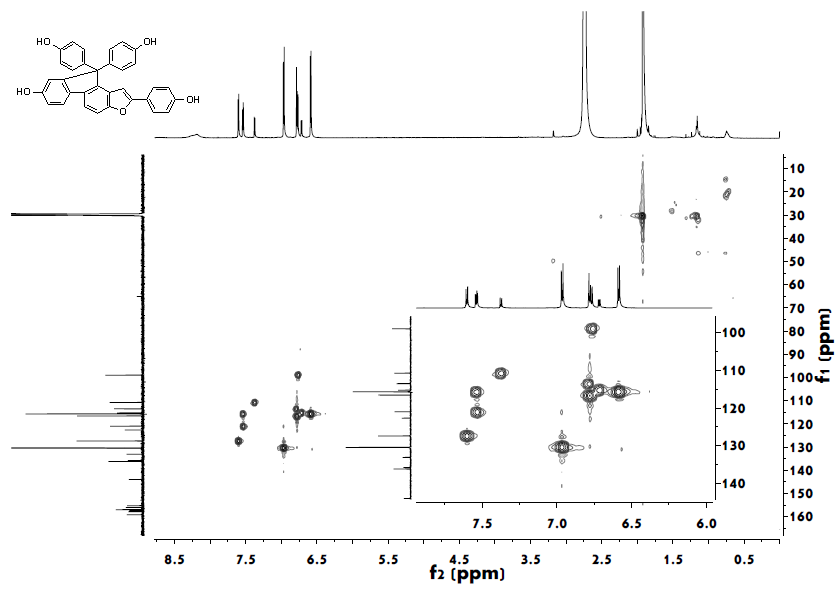


**Figure S6.** HMBC spectrum spectrum of isoselagintamarlin A (**1**)


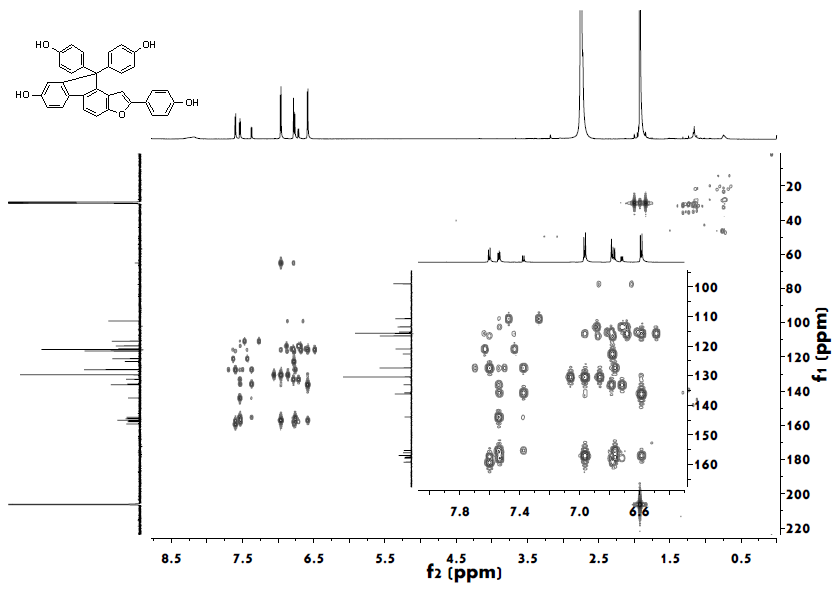


Figure **S7.** UV spectrum of isoselagintamarlin A (**1**)


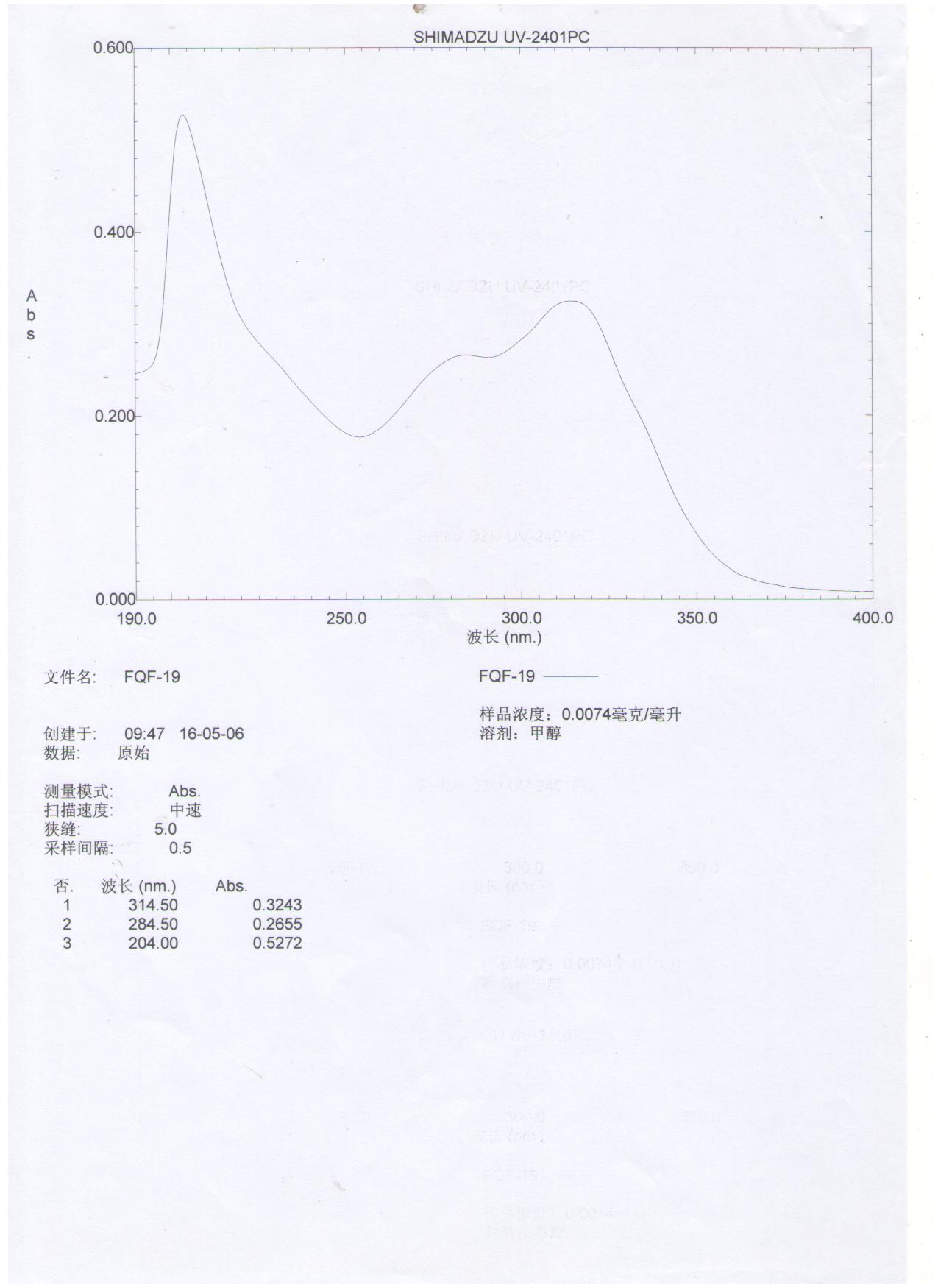


**Figure S8.** IR spectrum of isoselagintamarlin A (**1**)


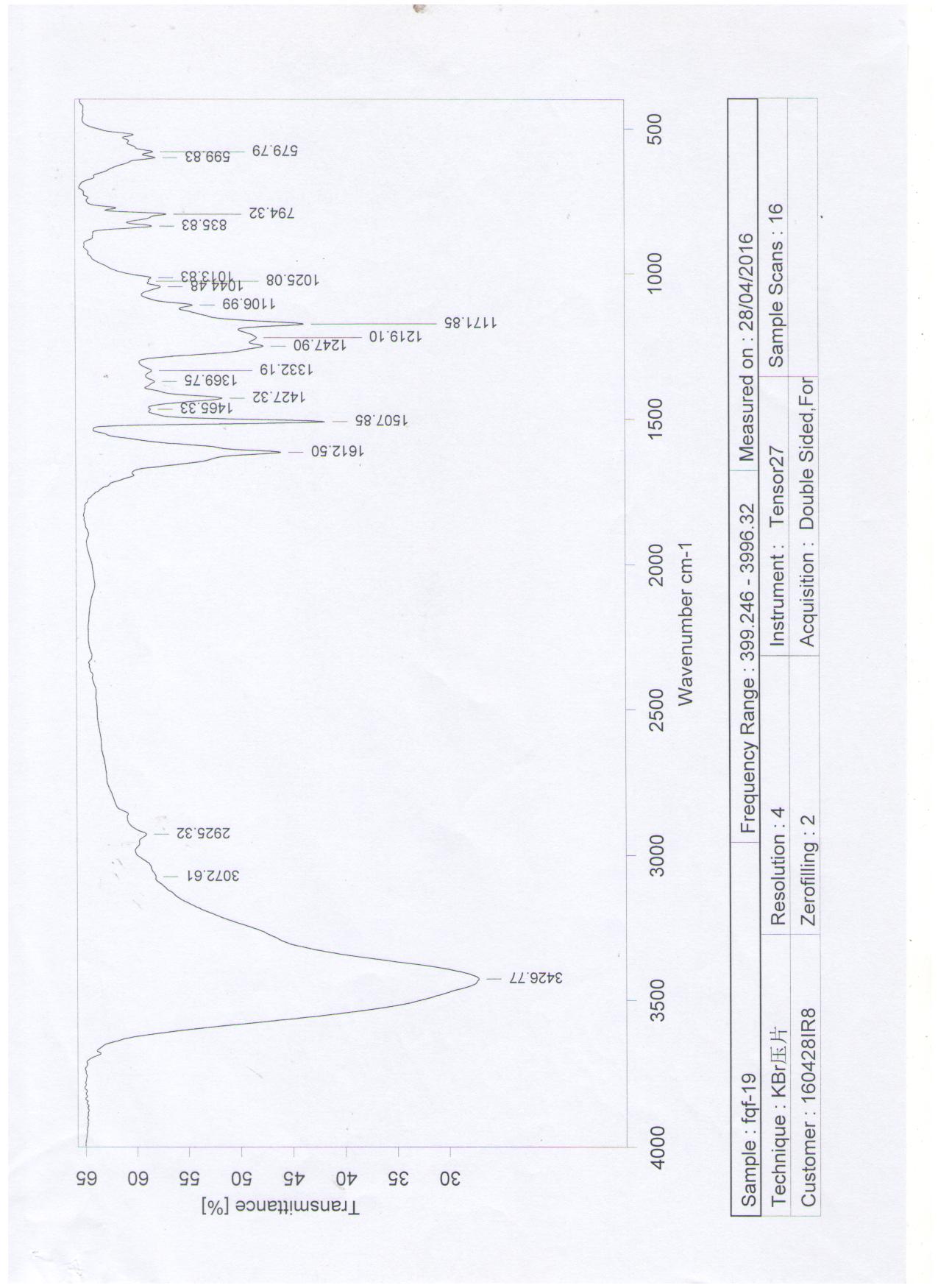


**Figure S9** .HR-EI-MS of isoselagintamarlin A (**1**)


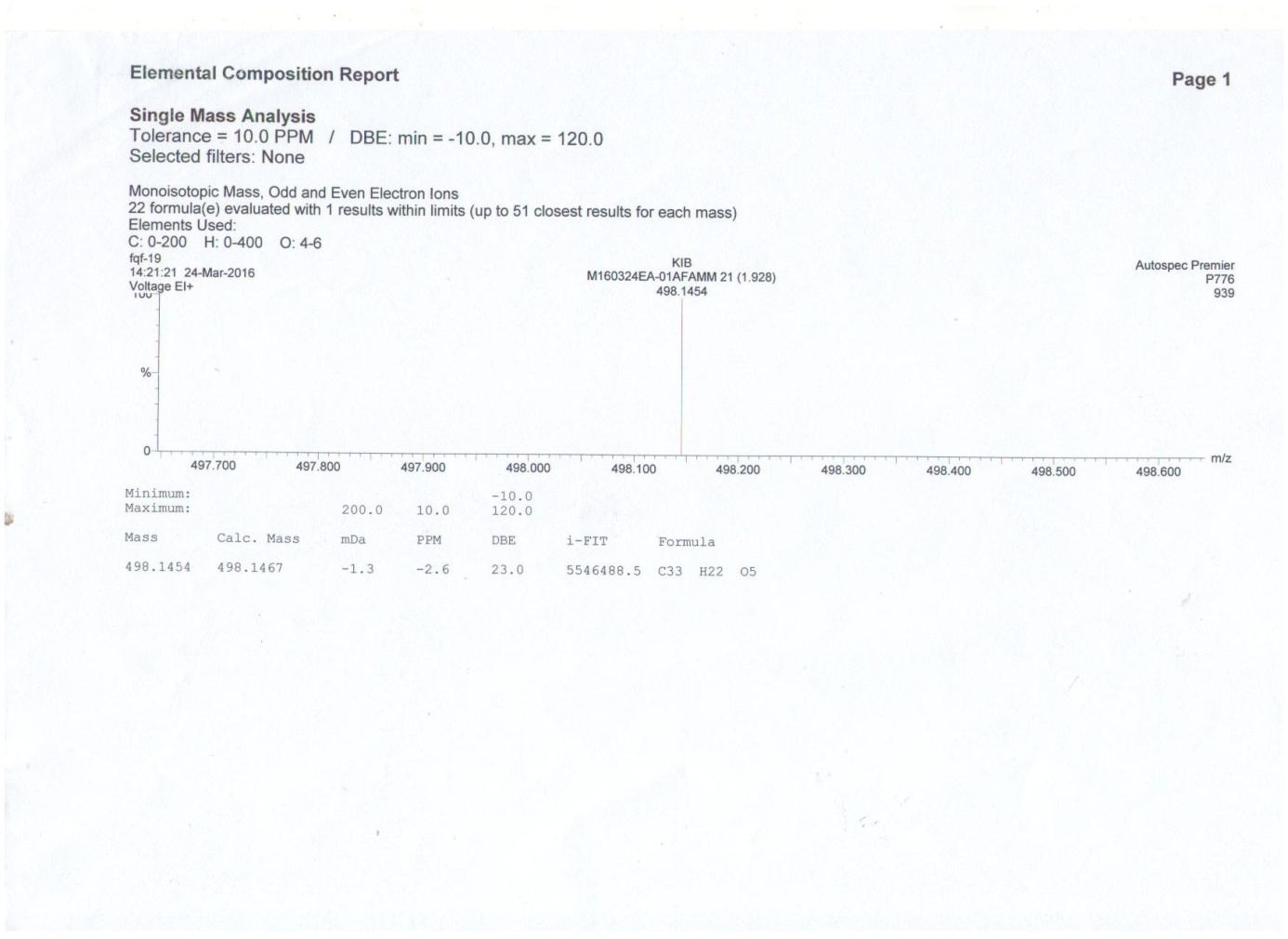


**Figure S10.** ^1^H NMR spectrum of *tetra*-acetylated selaginpulvilin A (**6**) in CDCl_3_ (600 MHz)


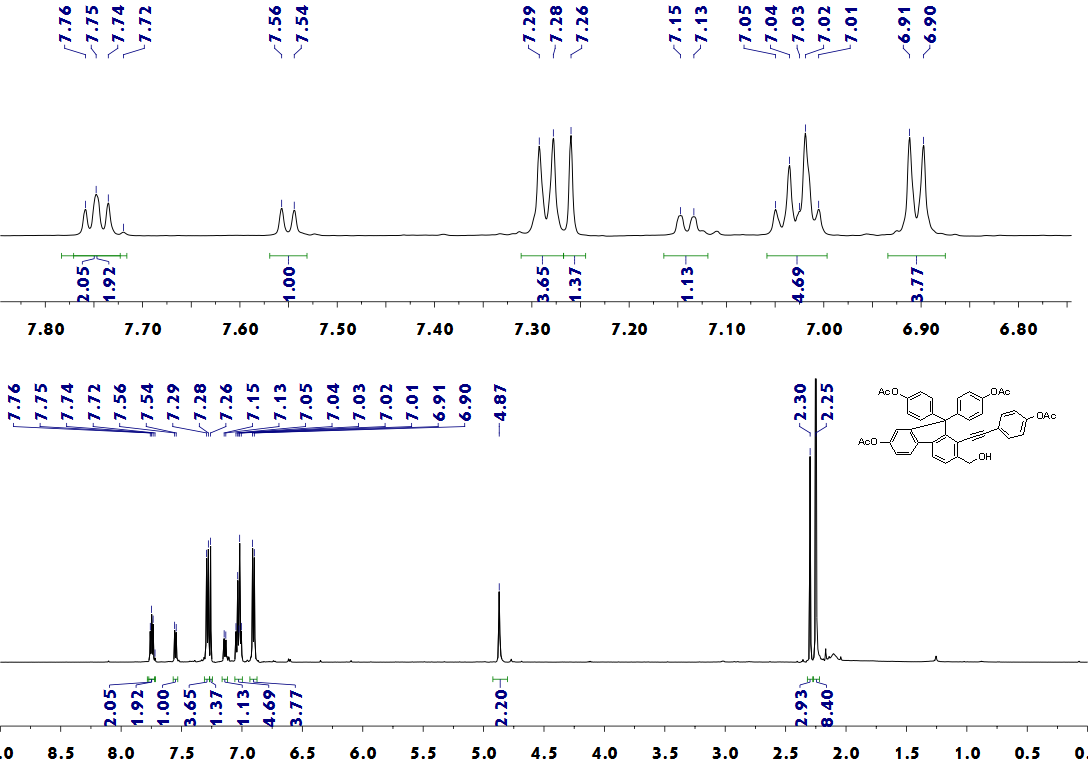


**Figure S11.** ^13^C NMR spectrum of *tetra*-acetylated selaginpulvilin A (**6**) in CDCl_3_ (150 MHz)


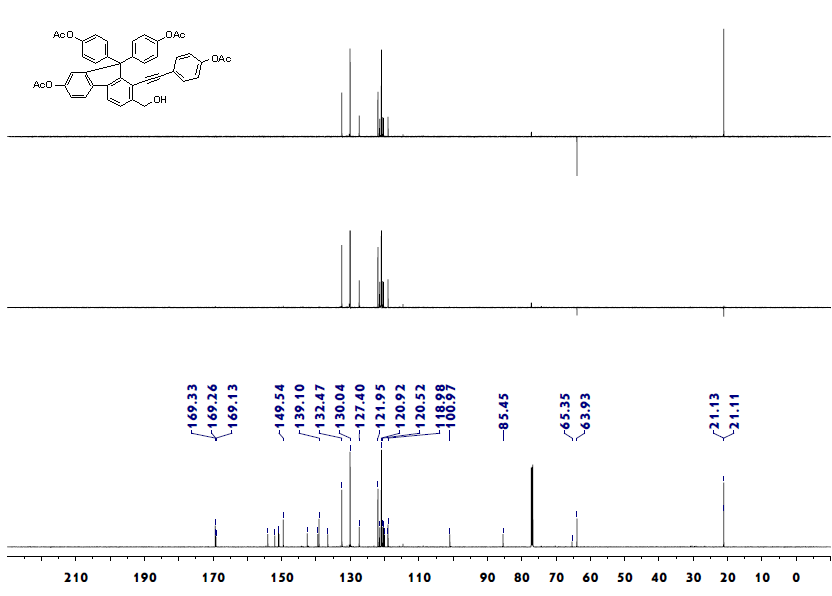


**Figure S12.** ^1^H^-1^H COSY of *tetra*-acetylated selaginpulvilin A (**6**)


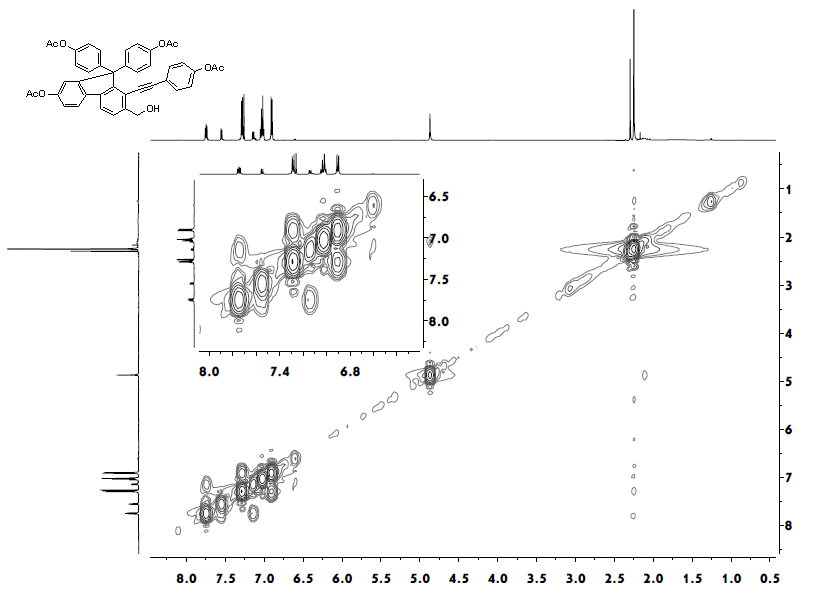


**Figure S13.** HSQC of *tetra*-acetylated selaginpulvilin A (**6**)


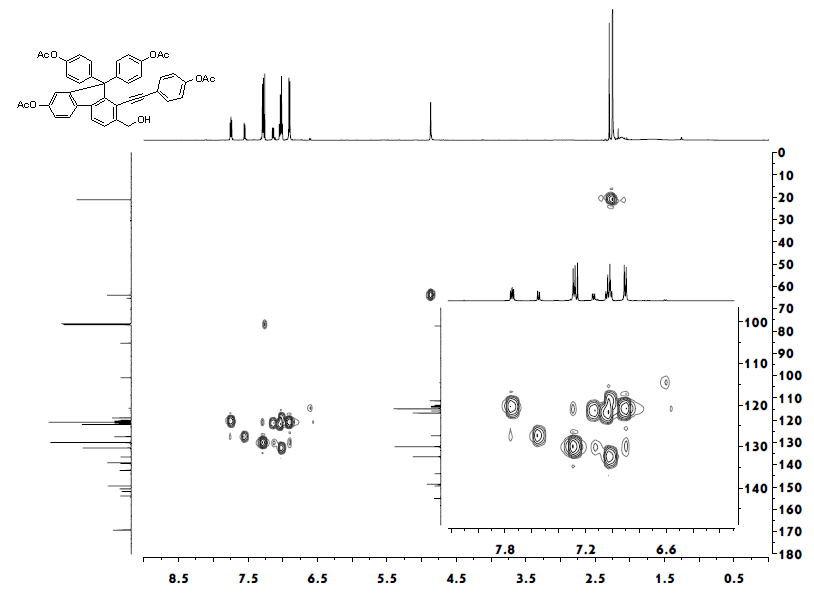


**Figure S14.** HMBC of *tetra*-acetylated selaginpulvilin A (**6**)


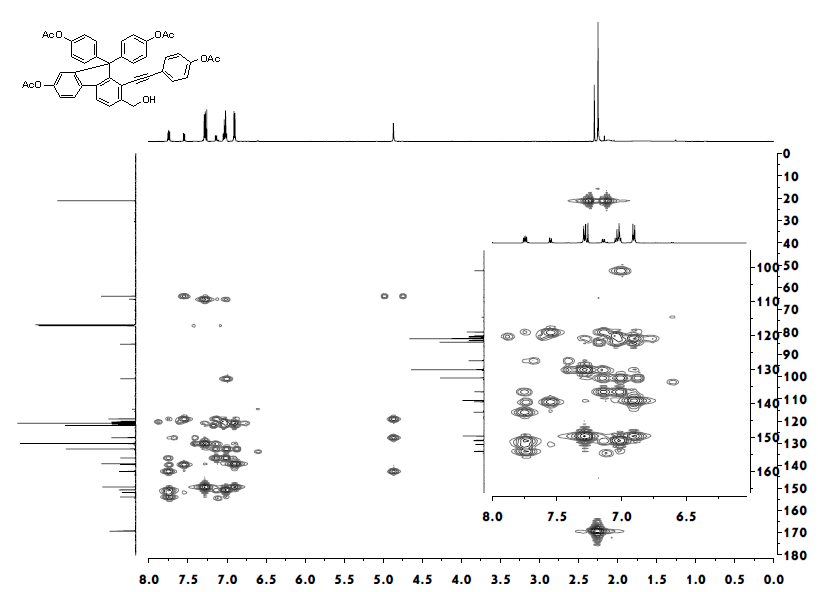


**Figure S15.** HR-ESI-MS of *tetra*-acetylated selaginpulvilin A (**6**)


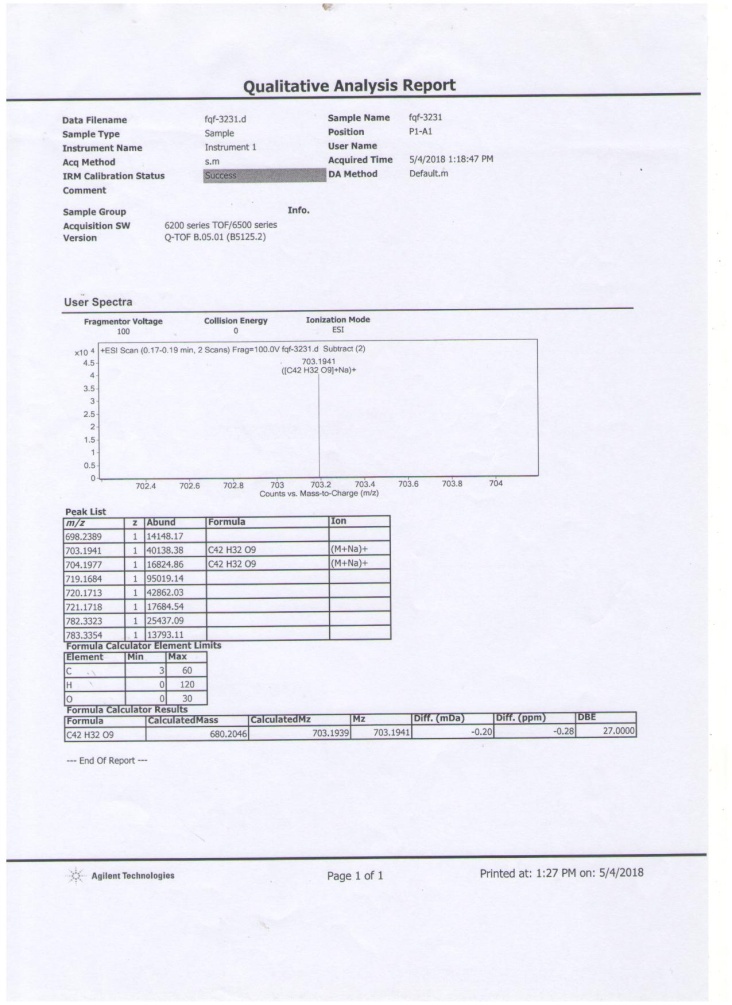


**Figure S16.** ^1^H NMR of *tetra*-acetylated selaginpulvilin B (**7**) in CDCl_3_ (600MHz)


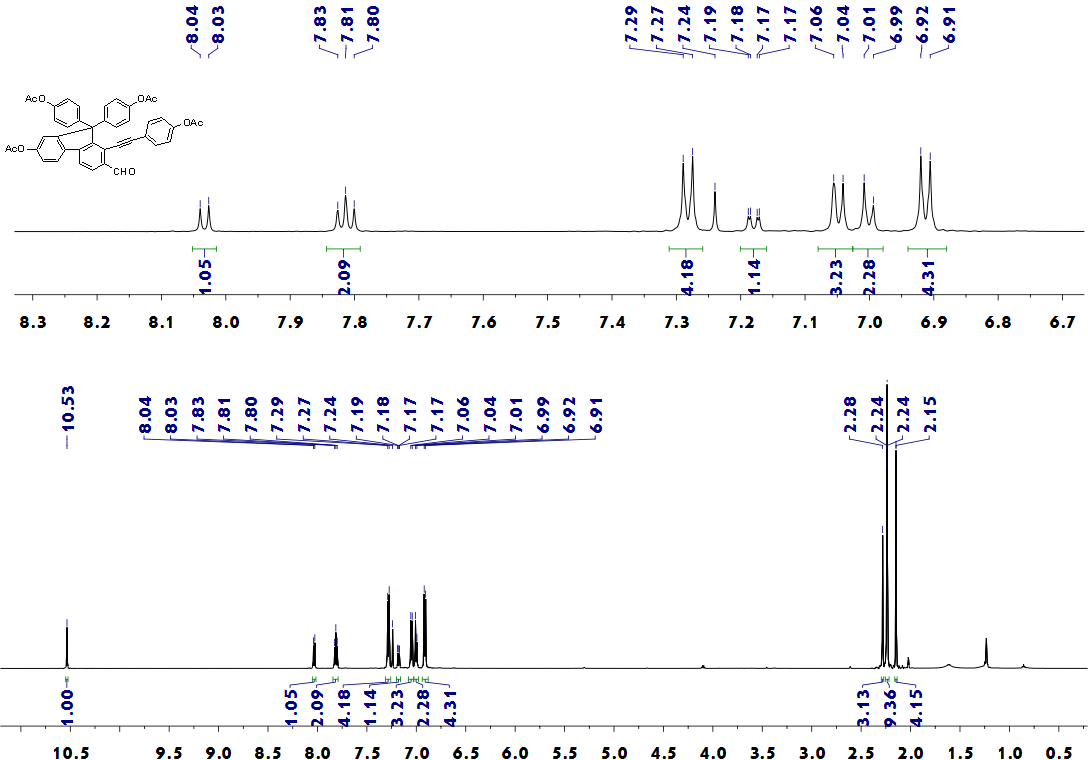


**Figure S17.** ^13^C NMR of *tetra*-acetylated selaginpulvilin B (**7**) in CDCl_3_ (150 MHz)


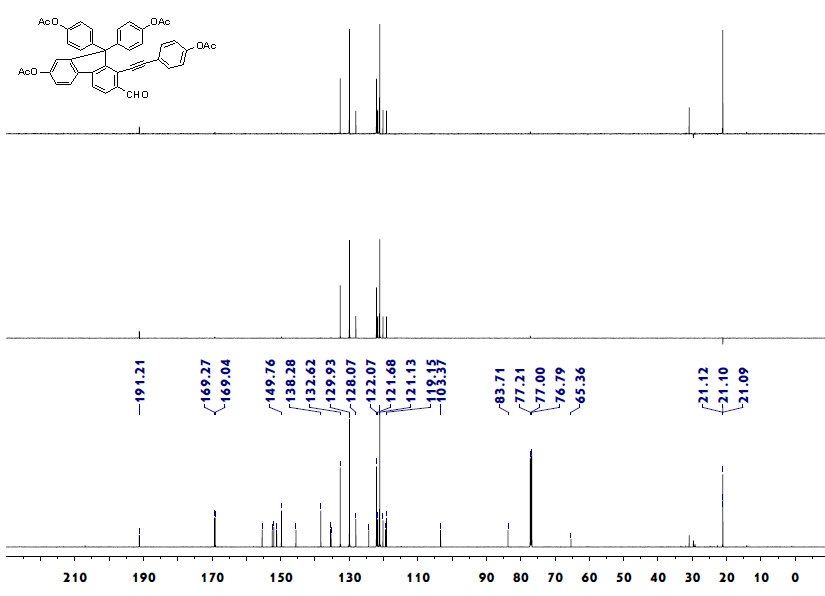


**Figure S18.** ^1^H-^1^H COSY of *tetra*-acetylated selaginpulvilin B (**7**)


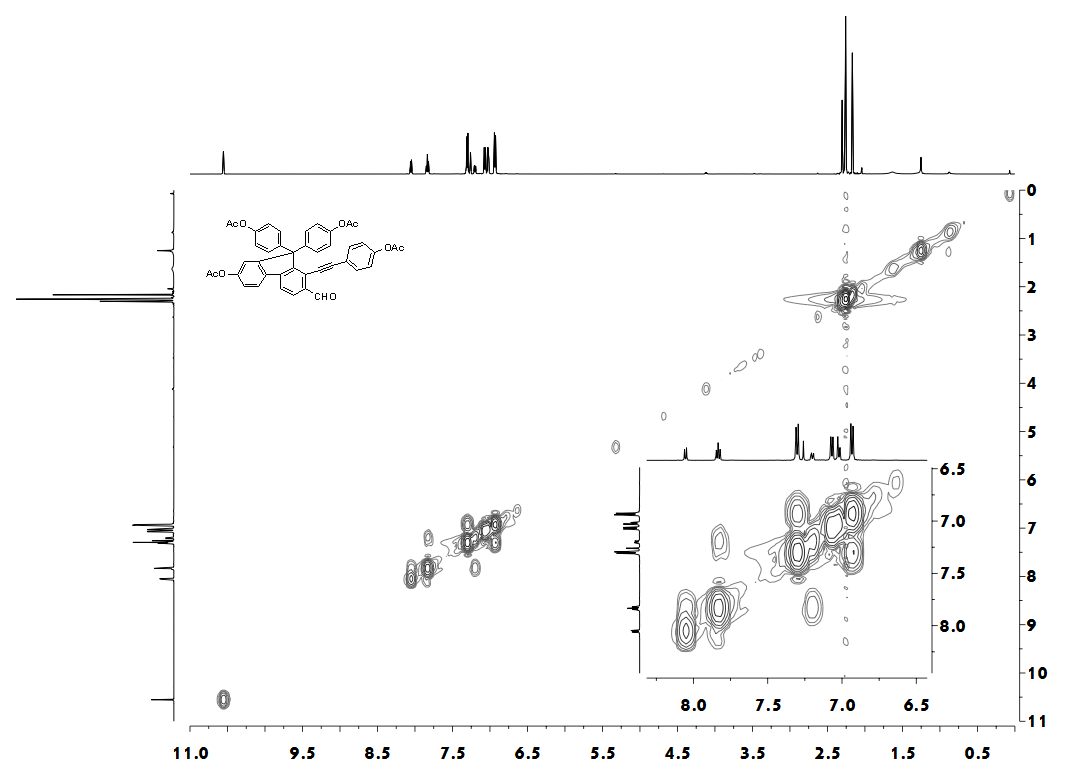


**Figure S19.** HSQC of *tetra*-acetylated selaginpulvilin B (**7**)


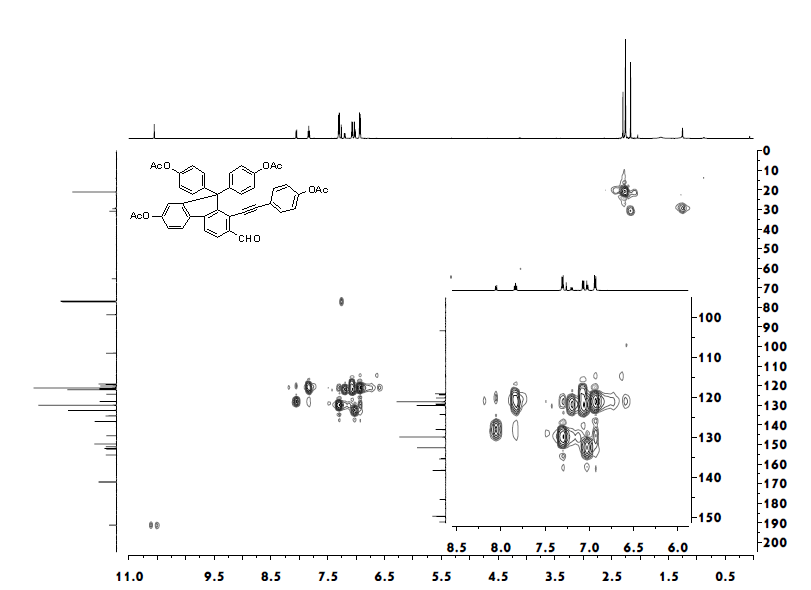


**Figure S20.** HMBC of *tetra*-acetylated selaginpulvilin B (**7**)


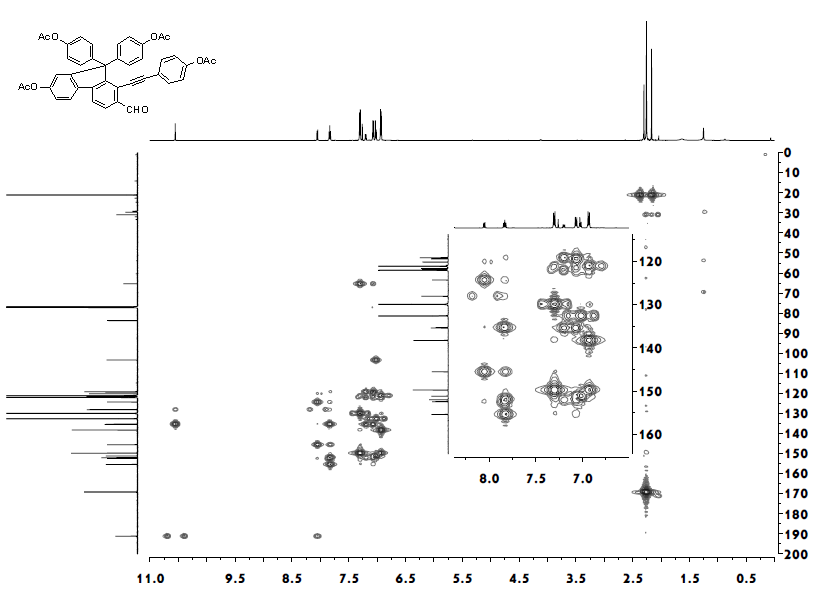


**Figure S21.** HR-ESI-MS of *tetra*-acetylated selaginpulvilin B (**7**)


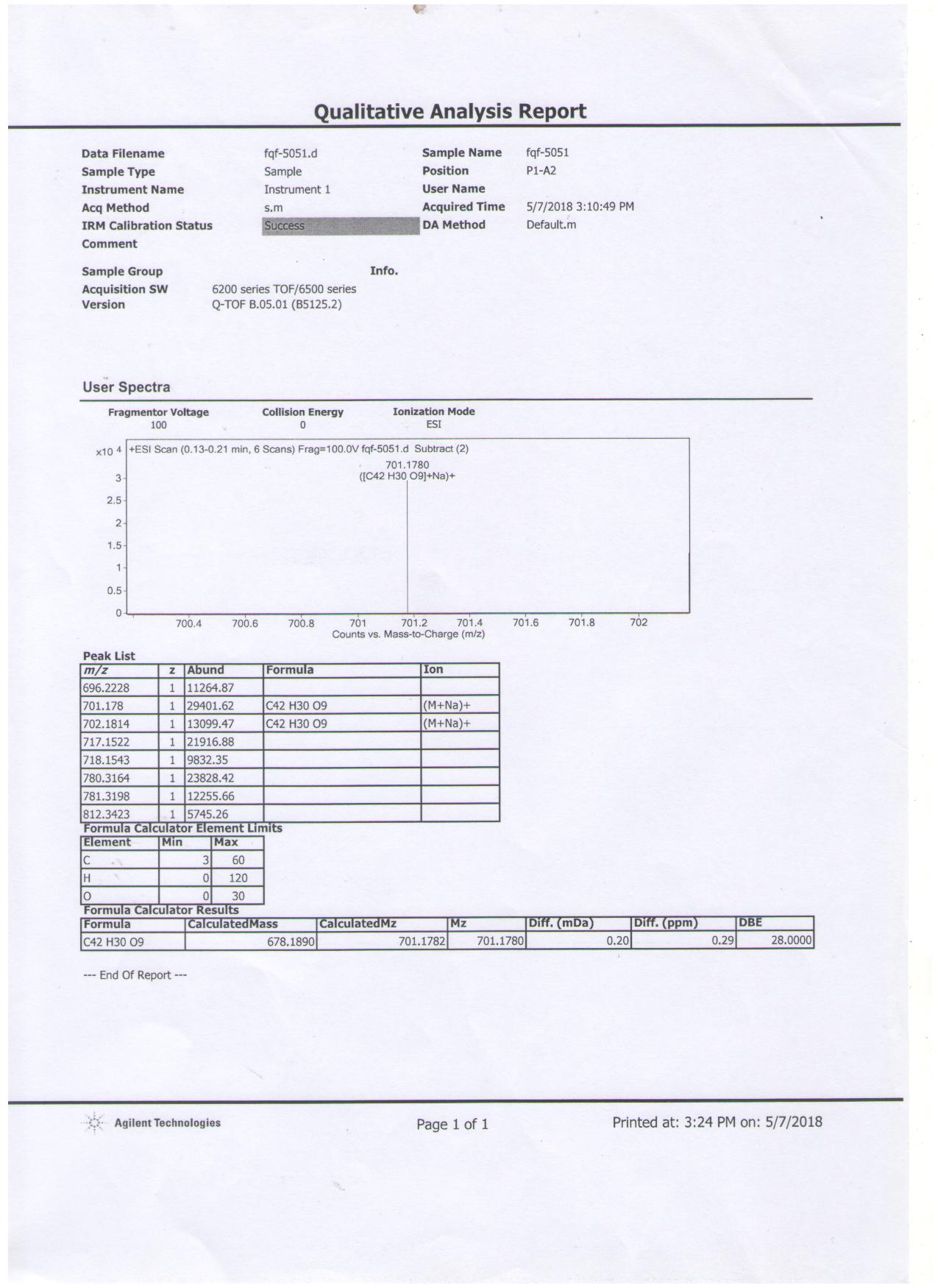


**Figure S22.** ^1^H NMR of *tetra*-acetylated selaginpulvilin J (**8**) in CDCl_3_ (600 MHz)


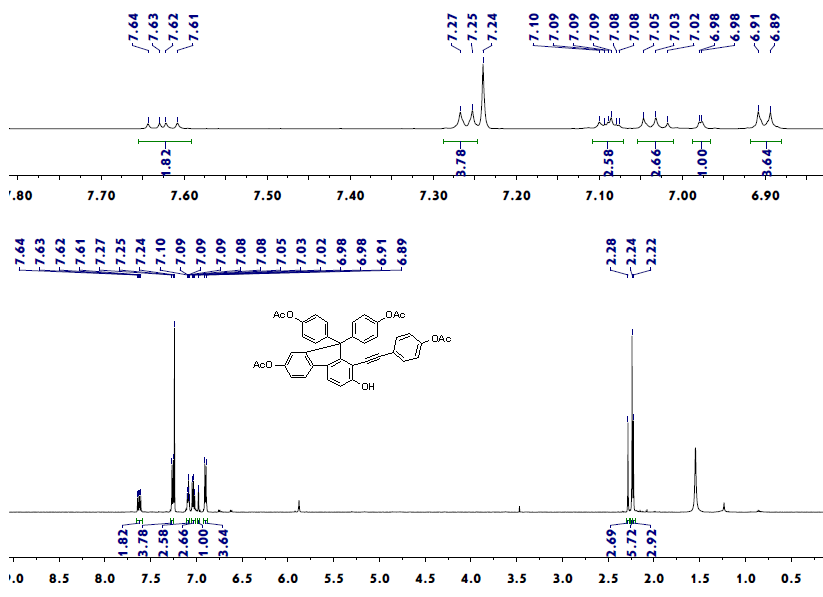


**Figure S23.** ^13^C NMR of *tetra*-acetylated selaginpulvilin J (**8**) CDCl_3_ (150 MHz)


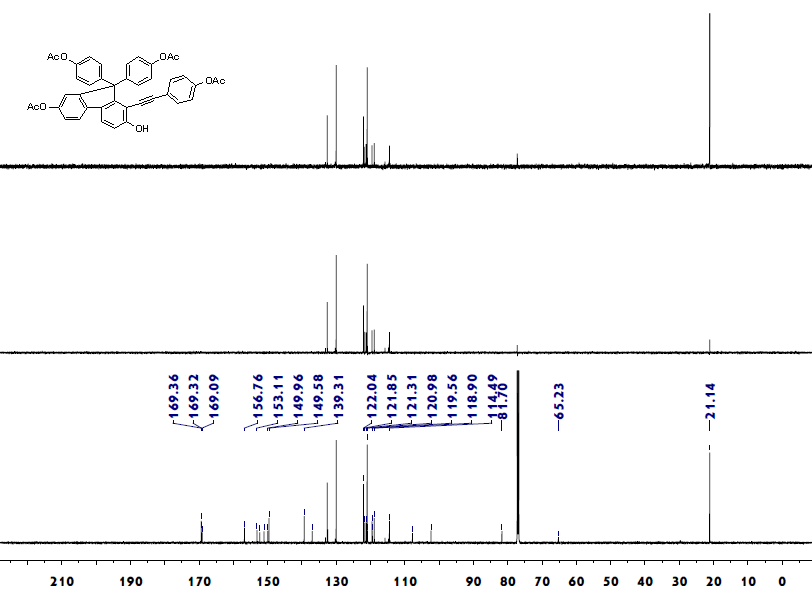


**Figure S24.** ^1^H^-1^H COSY of *tetra*-acetylated selaginpulvilin J (**8**)


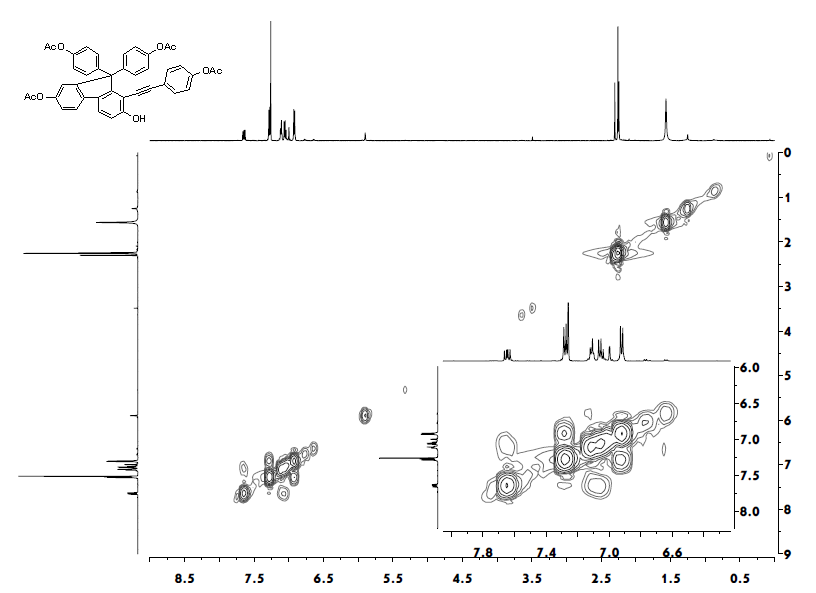


**Figure S25.** HSQC of *tetra*-acetylated selaginpulvilin J (**8**)


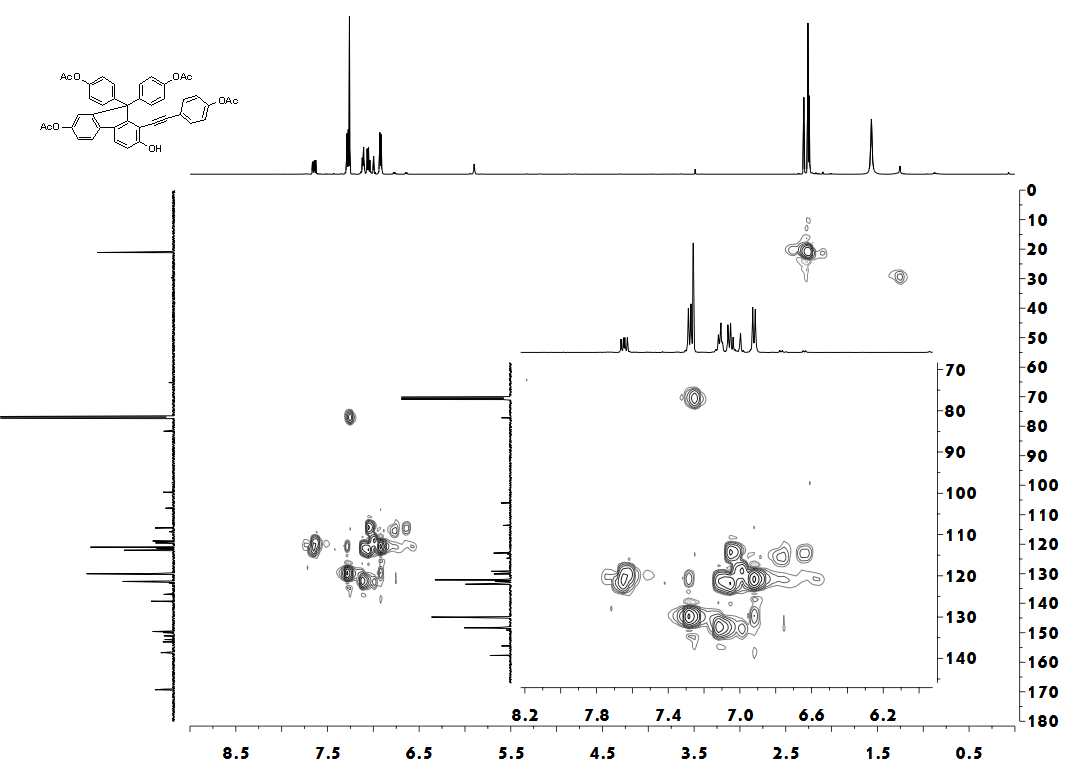


**Figure S26.** HMBC of *tetra*-acetylated selaginpulvilin J (**8**)


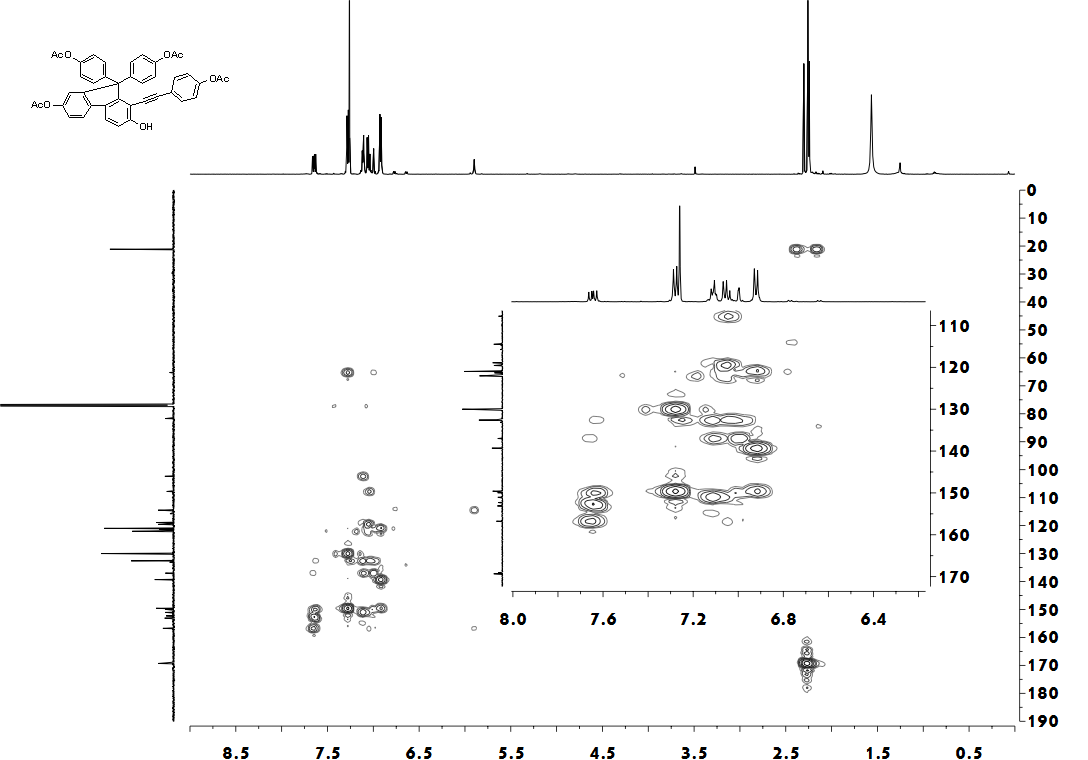


**Figure S27.** HR-ESI-MS of *tetra*-acetylated selaginpulvilin J (**8**)


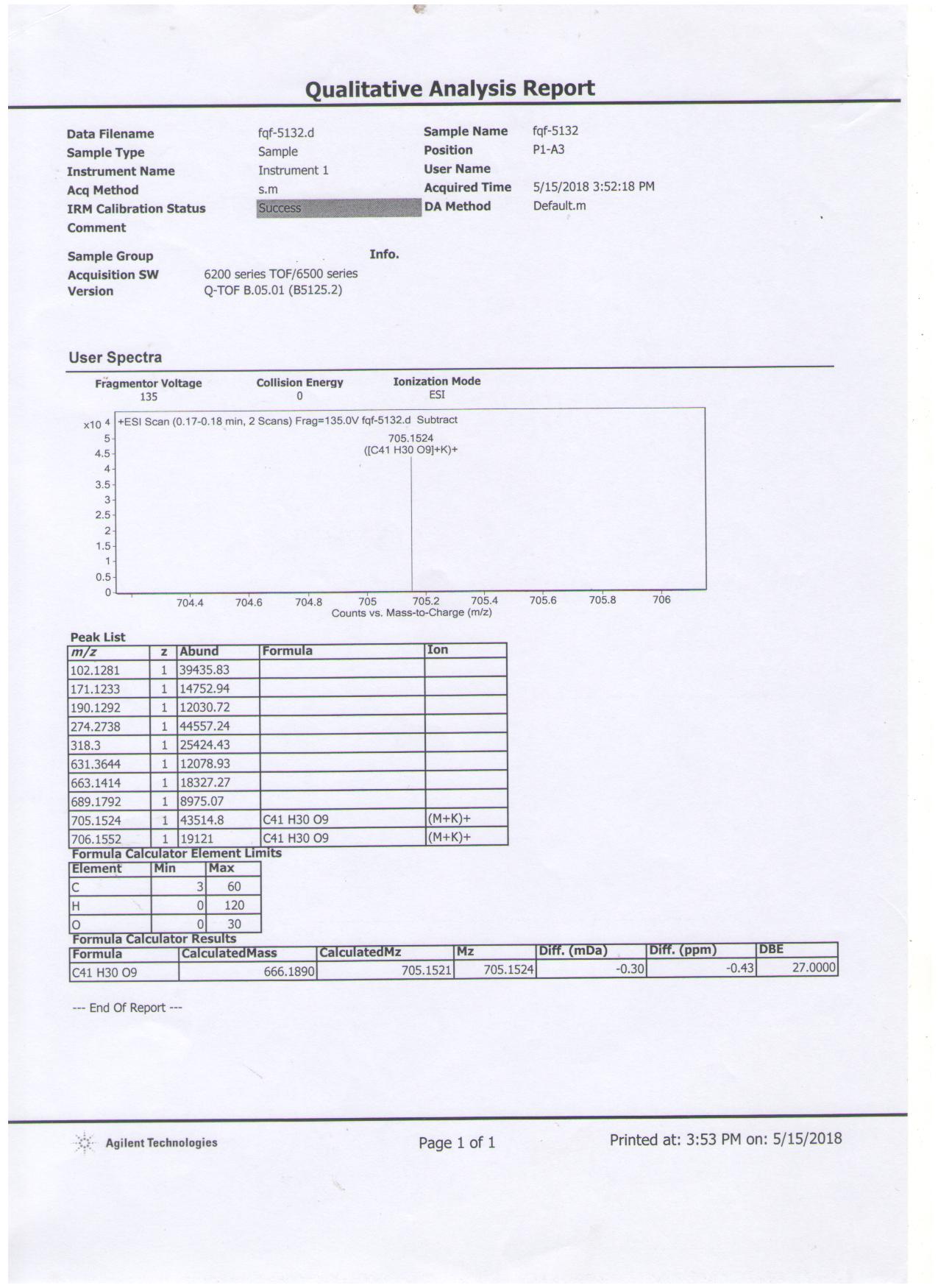


**Figure S28.** ^1^H NMR of *tetra*-acetylated isoselagintamarlin A (**9**) in acetone-*d*_6_ (600 MHz)


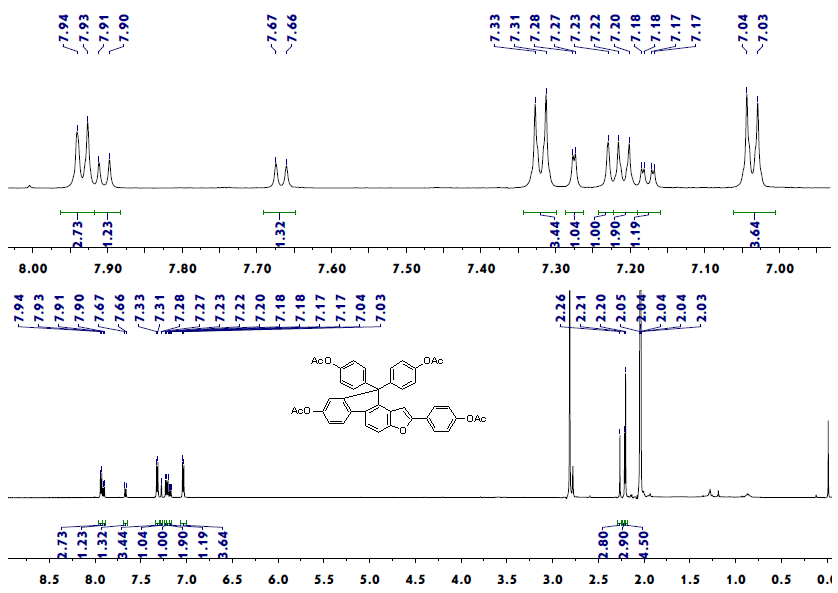


**Figure S29.** ^13^C NMR of *tetra*-acetylated isoselagintamarlin A (**9**) in acetone-*d*_6_ (150 MHz)


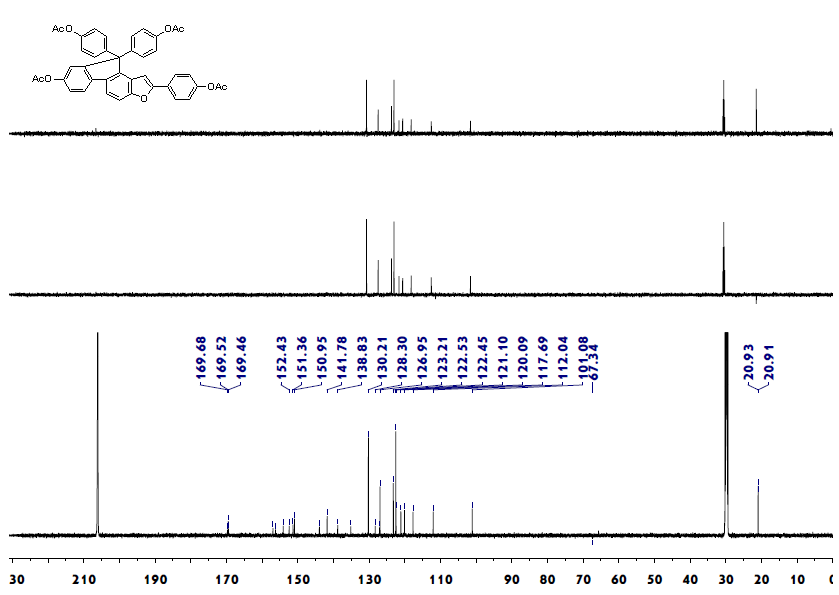


**Figure S30.** ^1^H^-1^H COSY NMR of *tetra*-acetylated isoselagintamarlin A (**9**)


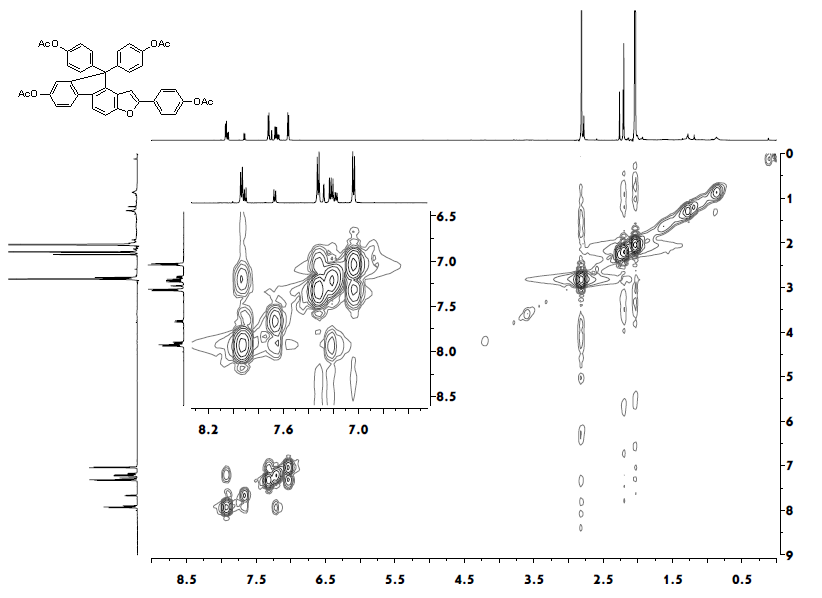


**Figure S31.** HSQC of *tetra*-acetylated isoselagintamarlin A (**9**)


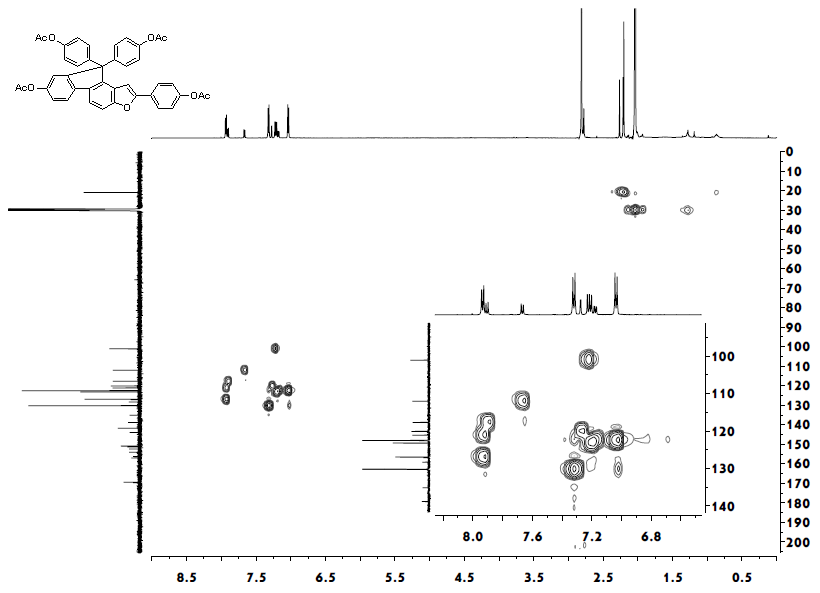


**Figure S32.** HMBC of *tetra*-acetylated isoselagintamarlin A (**9**)


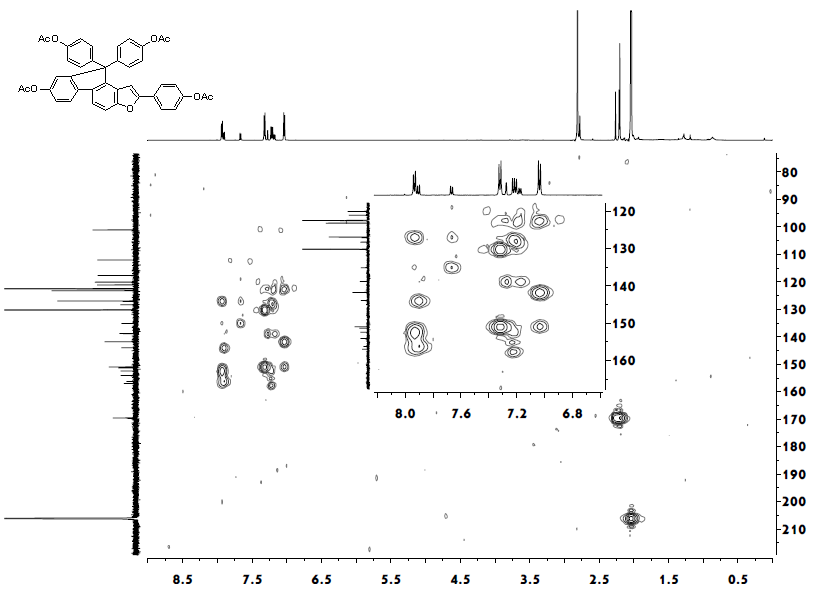


**Figure S33.** HR-ESI-MS of *tetra*-acetylated isoselagintamarlin A (**9**)


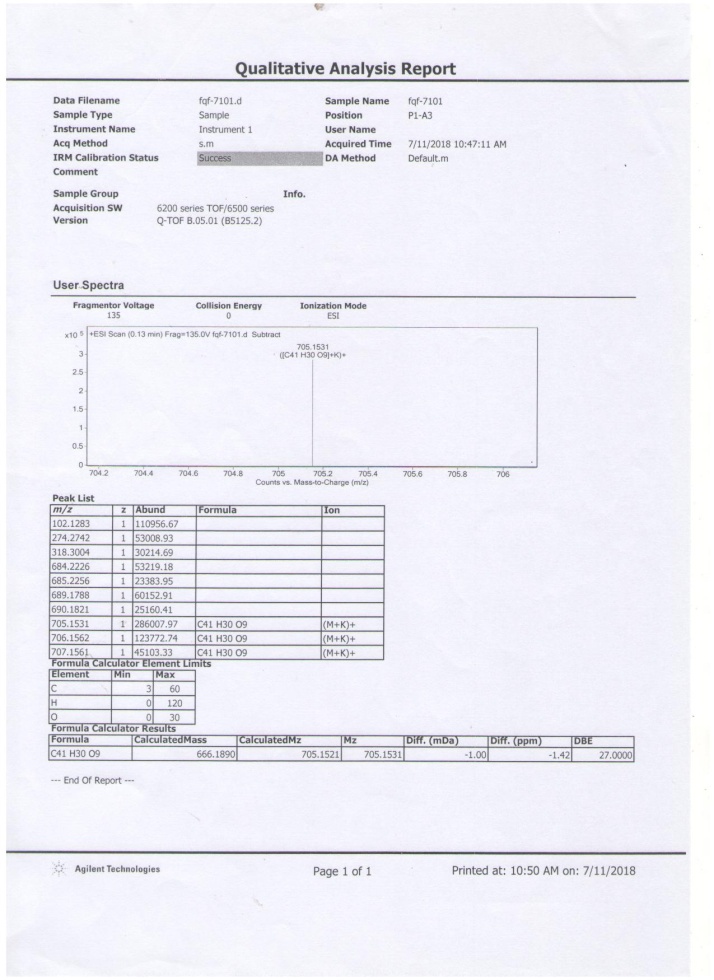


**Figure S34.** ^1^H NMR of isoselagintamarlin A (**1**) (synthetic product) in acetone-*d*_6_(800 MHz)


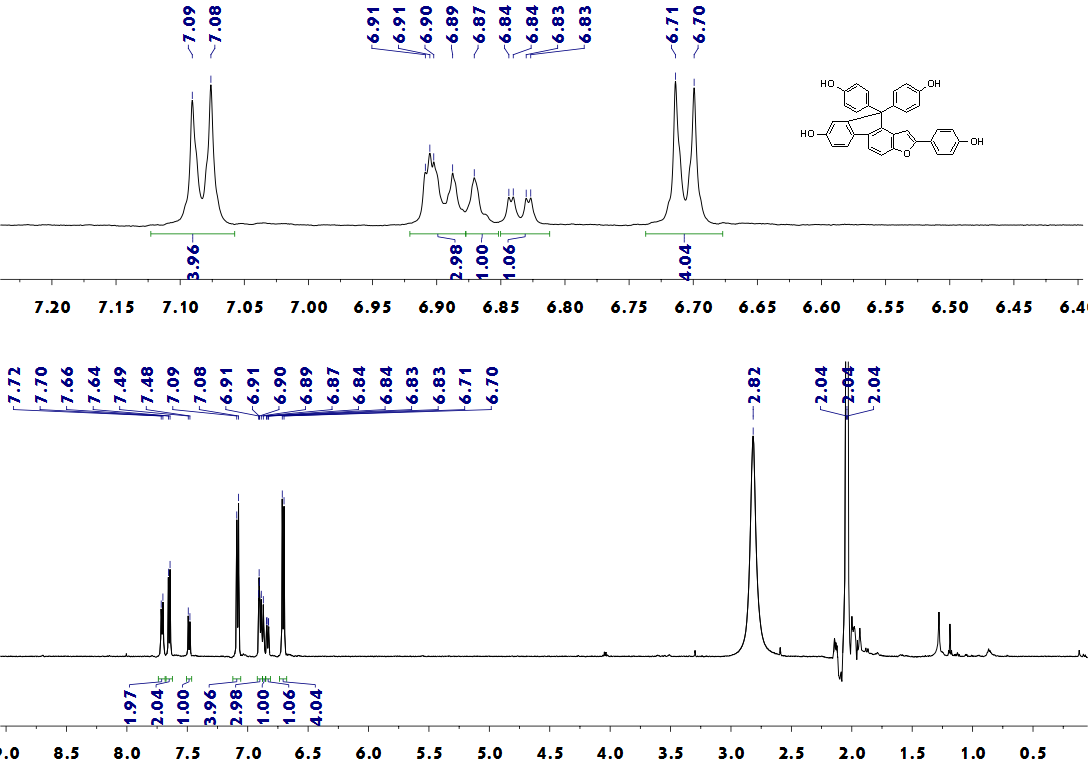


**Figure S35.** ^13^C NMR of isoselagintamarlin A (**1**) (synthetic product) in acetone-*d*_6_(200 MHz)


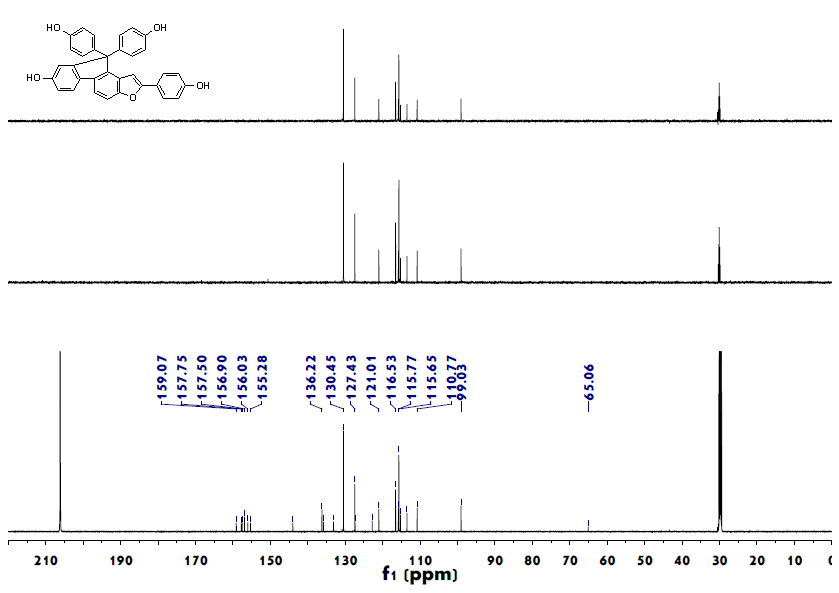


**Figure S36.** HR-ESI-MS of isoselagintamarlin A (**1**) (synthetic product)


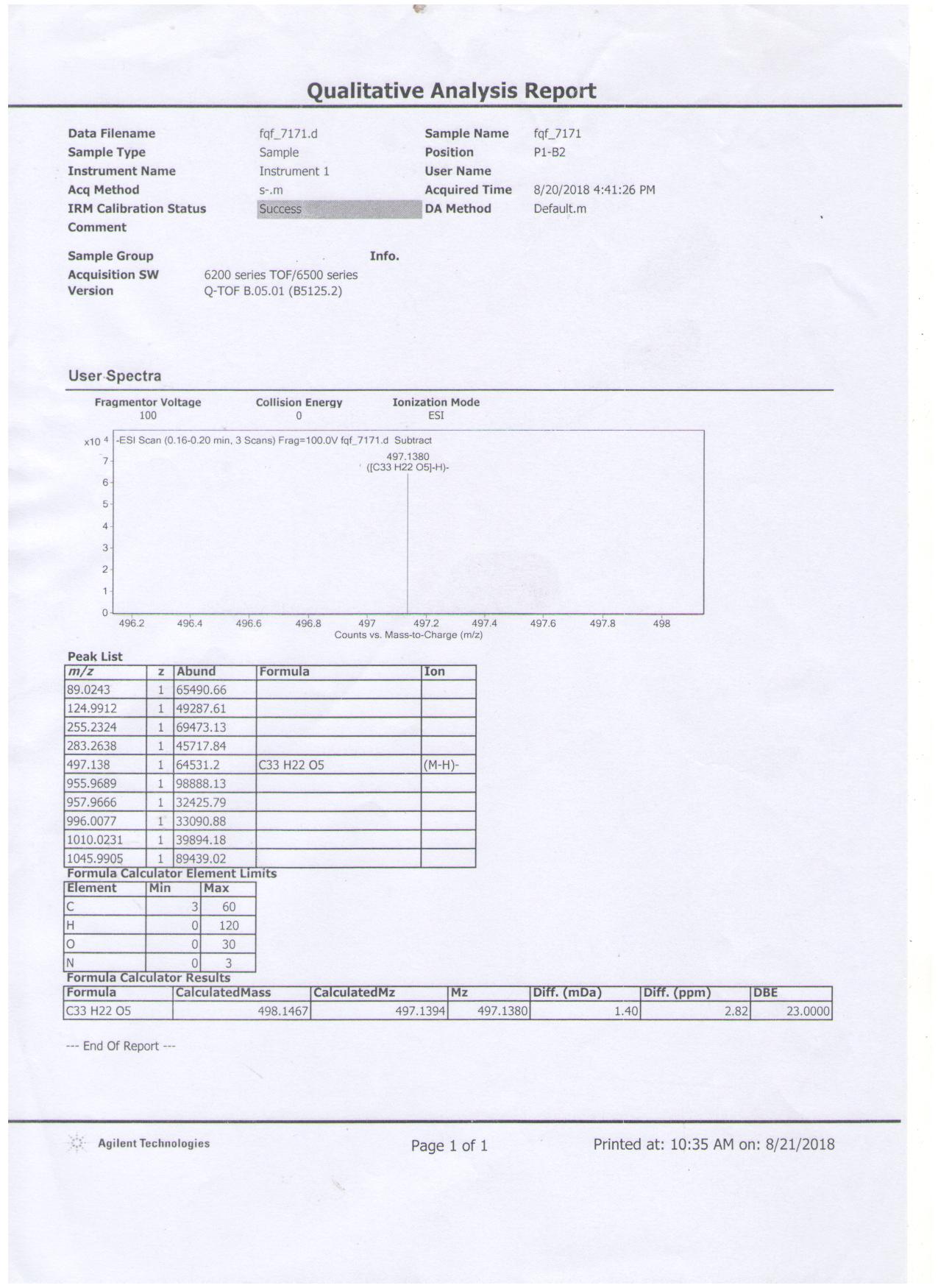

Supplement: Supplementary file 1 — Supplementary material 1 (DOCX 3034 kb) [file 13659_2018_195_MOESM1_ESM.docx]
